# Supplementary material for: Local and substrate-specific S-palmitoylation determines subcellular localization of Gαo
Source: Nat Commun. 2022 Apr 19;13:2072. doi: 10.1038/s41467-022-29685-8 (PMC9018777; doi:10.1038/s41467-022-29685-8)
Supplement: Supplementary file 1 — Supplementary Information [file 41467_2022_29685_MOESM1_ESM.pdf]

SUPPLEMENTARY INFORMATION FOR:

Local and substrate-specific S-palmitoylation determines subcellular localization of Gαo

Gonzalo P. Solis[1]*, Arghavan Kazemzadeh[1], Laurence Abrami[2], Jana Valnohova[1], Cecilia Alvarez[3], F. Gisou van der Goot[2] and Vladimir L. Katanaev[1,4]*

[1]Translational Research Center in Oncohaematology, Department of Cell Physiology and Metabolism, Faculty of Medicine, University of Geneva, Geneva, Switzerland;

[2]Global Health Institute, School of Life Sciences, EPFL, Lausanne, Switzerland;

[3]Centro de Investigaciones en Bioquímica Clínica e Inmunología (CIBICI-CONICET). Departamento de Bioquímica Clínica, Facultad de Ciencias Químicas, Universidad Nacional de Córdoba, Córdoba, Argentina;

[4]School of Biomedicine, Far Eastern Federal University, Vladivostok, Russia;

*Authors for correspondence: gonzalo.solis@unige.ch; vladimir.katanaev@unige.ch

Supplementary Methods

Supplementary Notes

Supplementary Figures 1-11

Supplementary References

Supplementary Methods

Screening of eukaryotic Gα subunits

To identify Gα subunit paralogs in eukaryotic genomes, the full-length amino acid sequence of Gαo was used for search queries in the NCBI protein Blast browser (blast.ncbi.nlm.nih.gov/Blast.cgi). Specifically, species with sequenced genomes were selected from Ensembl databases (ensembl.org) for vertebrates, plants, fungi, metazoa and protist. For the remaining phylogenetic clades, Gα paralogs were found as above and using the NCBI Taxonomy browser (ncbi.nlm.nih.gov/Taxonomy/Browser/wwwtax.cgi) as guide for clade classification. Sequences were manually curated and when annotated as partial were not considered further for this analysis. A total of 262 species were found to express Gα paralogs among the clades Amoebozoa, Discoba, Haptista, Metamonada, Opisthokonta, Sar, and Viridiplantae (Supplementary Data 1). Logo representation of the unique sequences selected for the different Gαo-Nt^7 categories (Supplementary Data 2) was done using the WebLogo application[1] (weblogo.berkeley.edu) and further edited with Inkscape v1.0.2 (inkscape.org).

Supplementary Notes

The eukaryotic Gα-Nt^7

To determine how much diversity is present in Nt^7 among eukaryotic Gα subunits, we searched for Gα sequences containing an N-terminal heptapeptide with the signature M^{1}GXXX[ACGST]X^7, with at least one Cys at positions 3-5 or 7. Of note, we did not find any Gα subunit orthologue in the clades Ancyromonadida, Apusozoa, Breviatea, Cryptophyceae, CRuMs, Glaucocystophyceae, Malawimonadidae, and Rhodophyta (red algae). We found, however, Gα subunits in abundance in Amoebozoa, Discoba, Haptista, Metamonada, Opisthokonta, Sar, and Viridiplantae. Overall, we identified more than 800 sequences containing the desired Nt^7 signature (Supplementary Data 1), and uncovered some interesting features. For instance, no Gα subunits was found with a Cys occupying position 7. On the other hand, we observed Cys throughout positions 3-5, with Cys3 as the most common case with just over half of the sequences. Cys4 follows as the second most frequent with one third of all cases, whereas only ~6% of Gα subunits contain a Cys5. The vast majority of Gα subunits containing a Cys5 are from plants (~65%) and Amoebozoa (~25%). Curiously, Cys5 is not present in Fungi and is almost completely absent in Metazoa, with the genus *Caenorhabditis* as the sole identified exception (e.g. *C. elegans*; Supplementary Data 1). Less than 10% of all sequences present two Cys residues occupying positions 3-5, and they almost exclusively belong to fungal Gα subunits (Supplementary Data 1). A double Cys at positions 3 and 4 is by far the most frequent combination among them. No Gα sequence was found with a Cys occupying all three positions.

We asked whether consensus sequences might emerge from the Gα subunits among each category. As available databases are biased towards certain clades, we first selected unique hexapeptides with the signature M^1GXXX[ACGST]^6. When an identical combination within positions 3-5 was found, we selected the sequence with the most frequent residue at position 7 for this analysis. Thus, we identified 88 unique combinations for Cys3, 75 for Cys4, and 13 for Cys5 (Supplementary Data 2). As seen in the corresponding Logo representations, there is no obvious Gα-Nt^7 consensus for the Cys3 and Cys4 categories (Supplementary Fig. 3a,b), whereas the sequence MGSLCSR emerges for Cys5 (Supplementary Fig. 3c). Plotting the amino acids by their polarity groups showed a common characteristic among categories: positively and even more so negatively charged amino acids are strongly underrepresented at positions 3-5 compared to a random distribution, or even completely absent as in the Cys5 category (Supplementary Fig. 3a-d). One can speculate that charged amino acids might interfere with at least one of the lipid modifications of Gα-Nt^7. The most recent recognition motif for NMTs – M^1G[^DEFRWY]X[^DEKR][ACGST][KR]^7 – denotes that positive and negative residues are not favored at certain positions[2]. Accordingly, N-myristoylation of Gαo-Nt^7-GFP appeared strongly impaired in N2a cells when different combinations of Arg, Asp, Glu, or Lys were introduced at positions 4 and 5 (Supplementary Fig. 3e). A Gαo-Nt^7-GFP mutant with a His occupying position 5 showed a near normal membrane association regardless of the presence of a positive or negative residue at position 4 (Supplementary Fig. 3f). The dissimilar localization of these Gαo-Nt^7 mutants was not due to major variations in their expression levels (Supplementary Fig. 3g,h). Thus, N-myristoylation of Gα-Nt^7 of the Cys3 category appears optimal when Arg, Asp, Glu, or Lys residues are excluded from position 5, in total agreement with the substrate recognition motif described by Castrec *et al*[2].

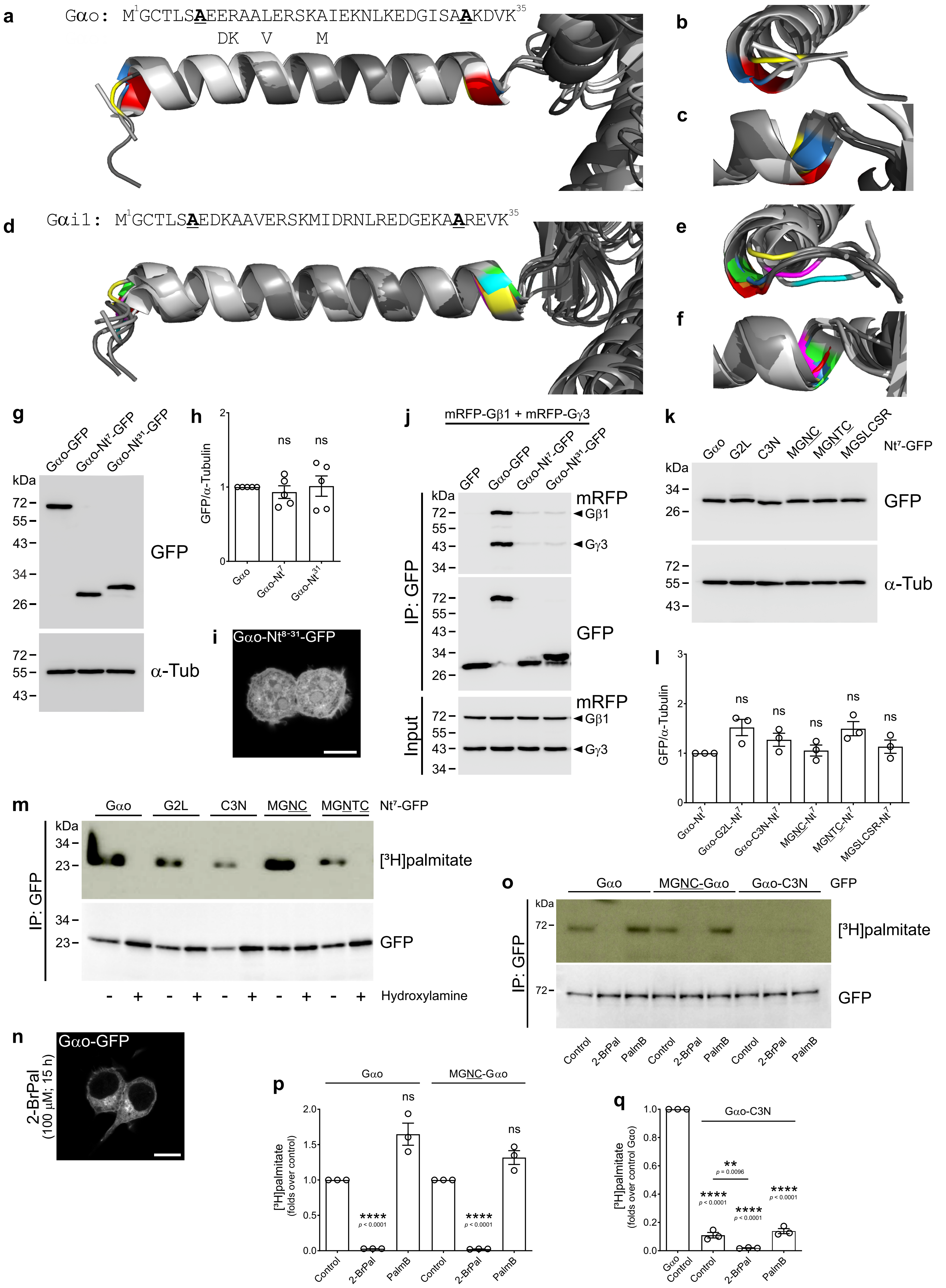

a
Gαo: M1GCTLSAEERAALERSKAIEKNLKEDGISAAKDVK35
DK V M
b
c
d
Gαi1: M1GCTLSAEDKAAVERSKMIDRNLREDGEKAAREVK35
e
f
g
Gαo-GFP Gαo-Nt7-GFP Gαo-Nt31-GFP
kDa
GFP
α-Tub
h
GFP/α-Tubulin
ns ns
Gαo Gαo-Nt7 Gαo-Nt31
i
Gαo-Nt8-31-GFP
j
mRFP-Gβ1 + mRFP-Gγ3
GFP Gαo-GFP Gαo-Nt7-GFP Gαo-Nt31-GFP
IP: GFP
Input
mRFP
◄Gβ1
◄Gγ3
GFP
k
Gαo G2L C3N MGNC MGNTC MGSLCSR Nt7-GFP
GFP
α-Tub
l
GFP/α-Tubulin
Gαo-Nt7 Gαo-G2L-Nt7 Gαo-C3N-Nt7 MGNC-Nt7 MGNTC-Nt7 MGSLCSR-Nt7
m
Gαo G2L C3N MGNC MGNTC Nt7-GFP
IP: GFP
[3H]palmitate
GFP
- + - + - + - + - + Hydroxylamine
n
Gαo-GFP
2-BrPal (100 μM; 15 h)
o
Gαo MGNC-Gαo Gαo-C3N GFP
IP: GFP
[3H]palmitate
GFP
Control 2-BrPal PalmB Control 2-BrPal PalmB Control 2-BrPal PalmB
p
Gαo MGNC-Gαo
[3H]palmitate (folds over control)
**** p < 0.0001
Control 2-BrPal PalmB Control 2-BrPal PalmB
q
Gαo-C3N
[3H]palmitate (folds over control Gαo)
** p = 0.0096
**** p < 0.0001
Gαo Control Control 2-BrPal PalmB

Supplementary Figure 1. Gαo N-terminal region

a-**c**, Overlay of the N-terminal region of three solved Gαo structures (**a**). N-terminus (**b**) and α-helix end (**c**) are magnified. Ala7 and Ala31 are underlined in Gαo sequence and color-coded in the structures (PDB-IDs): red for 6g79[3], blue for 6k41[4], yellow for 6oik[5]. The 6oik structure contained four substitutions indicated below the sequence in (**a**). **d**-**f**, Overlay of seven solved Gαi1 structures (**d**). Magnification of N-terminus (**e**) and α-helix end (**f**). Ala7 and Ala31 underlined in the sequence and color-coded in the structures (PDB-IDs): green for 5kdo[6], cyan for 1gg2[7], pink for 6ddf[8], yellow for 6k42[4], orange for 6kpf[9], red for 6n4b[10], blue for 6osa[11]. **g**,**h**, Expression in N2a cells of Gαo-GFP, Gαo-Nt^{7}-GFP or Gαo-Nt^{31}-GFP. Anti-GFP antibody was used to detect Gαo constructs and α-Tubulin (α-Tub) served as loading control (**g**). Quantification of Gαo constructs expression (**h**). Data as mean ± s.e.m.; 5 independent experiments. **i**, Localization of Gαo-Nt$^{8\text{-}31}$-GFP in N2a cells. **j**, Immunoprecipitation (IP) of Gαo-GFP, Gαo-Nt^{7}-GFP, Gαo-Nt^{31}-GFP or GFP as control. Only Gαo-GFP efficiently co-precipitated mRFP-Gβ1γ3. Antibodies against GFP and mRFP were used for detection of Gαo constructs and Gβ1γ3, respectively. **k**,**l**, Expressing of Gαo-Nt^{7}-GFP wild-type and mutants, and the consensus MGSLCSR-Nt^{7}-GFP (**k**). Underlined letters indicate residues mutated in Gαo-Nt^{7}. Detection and Quantification as in (**g**,**l**). Data as the mean ± s.e.m.; 3 independent experiments. **m**, [^{3}H]palmitate radiolabeling of HeLa cells expressing Gαo-Nt^{7}-GFP wild-type and mutants. Immunoprecipitated GFP-fusions from control (-) and hydroxylamine (+) treated samples were analyzed by autoradiography ([^{3}H]palmitate) and anti-GFP antibody. A long exposure is shown for visualization of weakly labelled constructs. **n**, Gαo-GFP localization in N2a cells treated with 2-bromopalmitate (2-BrPal). **o**-**q**, [^{3}H]palmitate radiolabeling of Gαo-GFP, MGNC-Gαo-GFP, and Gαo-C3N-GFP (**o**). GFP-fusions from control, 2-BrPal and Palmostatin B (PalmB) treated cells were analyzed as in (**m**). [^{3}H]palmitate incorporation to constructs normalized to corresponding control (**p**) or Gαo (**q**). Data as mean ± s.e.m.; 3 independent experiments. **i**,**n**, Scale bars, 10 µm. *P* values were determined using one-sample *t*-test for **h**,**l**,**p**,**q** and two-sided unpaired *t*-test for **q**. ns: not significant. Source data are provided as a Source Data file.

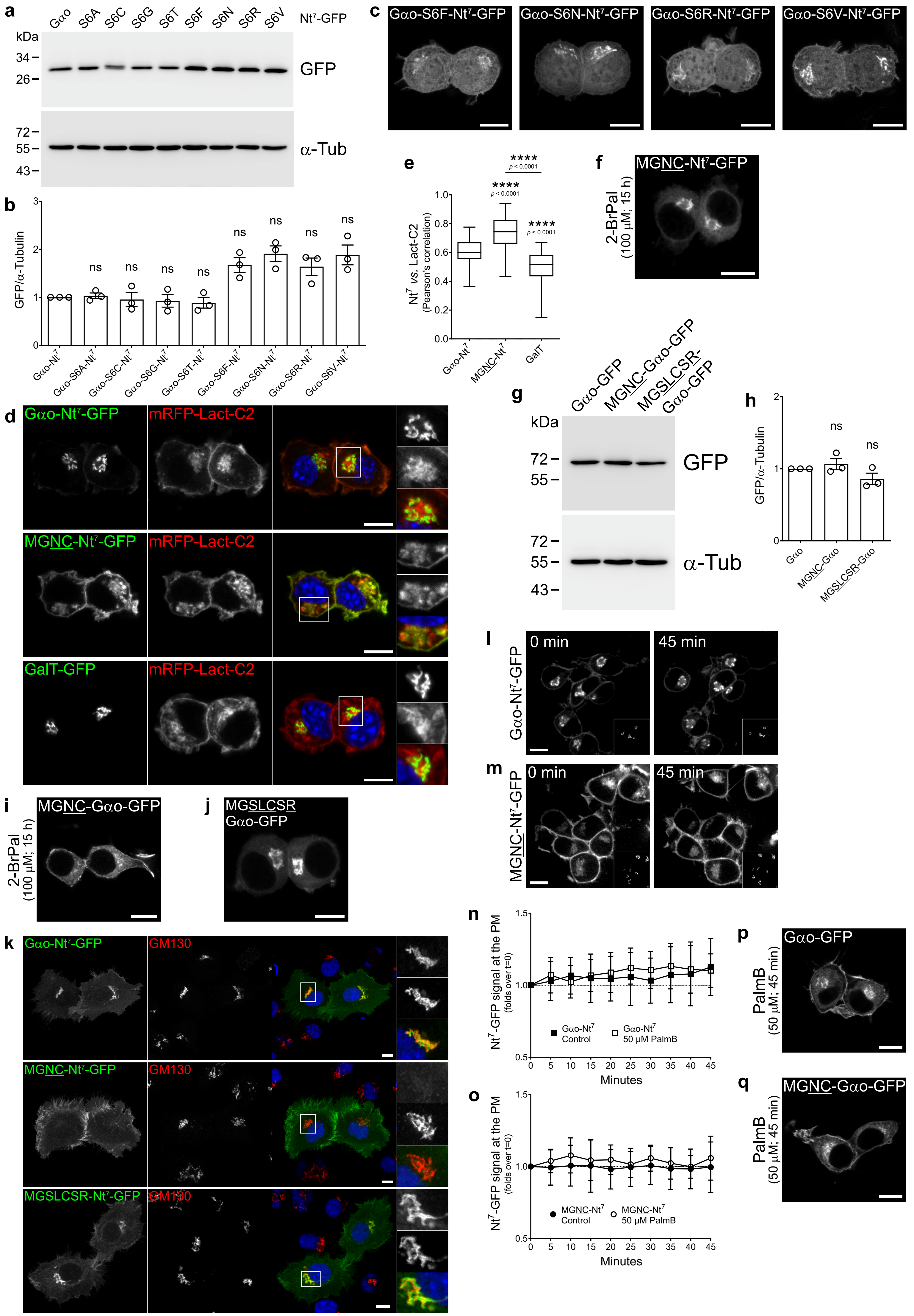

a
Gαo S6A S6C S6G S6T S6F S6N S6R S6V Nt⁷-GFP
kDa
34
26
GFP
72
55
43
α-Tub
b
GFP/α-Tubulin
ns
Gαo-Nt7
Gαo-S6A-Nt7
Gαo-S6C-Nt7
Gαo-S6G-Nt7
Gαo-S6T-Nt7
Gαo-S6F-Nt7
Gαo-S6N-Nt7
Gαo-S6R-Nt7
Gαo-S6V-Nt7
c
Gαo-S6F-Nt⁷-GFP
Gαo-S6N-Nt⁷-GFP
Gαo-S6R-Nt⁷-GFP
Gαo-S6V-Nt⁷-GFP
e
Nt7 vs. Lact-C2 (Pearson's correlation)
****
p < 0.0001
Gαo-Nt7
MGNC-Nt7
GalT
f
MGNC-Nt⁷-GFP
2-BrPal (100 µM; 15 h)
g
Gαo-GFP
MGNC-Gαo-GFP
MGSLCSR-Gαo-GFP
kDa
72
55
GFP
43
α-Tub
h
GFP/α-Tubulin
ns
Gαo
MGNC-Gαo
MGSLCSR-Gαo
d
Gαo-Nt⁷-GFP
mRFP-Lact-C2
MGNC-Nt⁷-GFP
GalT-GFP
l
Gαo-Nt⁷-GFP
0 min
45 min
m
MGNC-Nt⁷-GFP
i
MGNC-Gαo-GFP
2-BrPal (100 µM; 15 h)
j
MGSLCSR Gαo-GFP
k
Gαo-Nt⁷-GFP
GM130
MGNC-Nt⁷-GFP
MGSLCSR-Nt⁷-GFP
n
Nt7-GFP signal at the PM (folds over t=0)
Gαo-Nt7 Control
Gαo-Nt7 50 µM PalmB
Minutes
o
MGNC-Nt7 Control
MGNC-Nt7 50 µM PalmB
p
Gαo-GFP
PalmB (50 µM; 45 min)
q
MGNC-Gαo-GFP

Supplementary Figure 2. Cys position within Nt^{7} sequences

a,**b**, Expression in N2a cells of Gαo-Nt^{7}-GFP wild-type and Ser6 mutants (**a**). Anti-GFP antibody was used to detect Gαo constructs and α-Tubulin (α-Tub) served as loading control. Quantification of Nt^{7} constructs expression (**b**). Data as mean ± s.e.m.; 3 independent experiments. **c**, Localization of Ser6 mutants of Gαo-Nt^{7}-GFP. **d**,**e**, Co-expression of mRFP-Lact-C2 with Gαo-Nt^{7}-GFP (top), MGNC-Nt^{7}-GFP (middle) or GalT-GFP (bottom) (**d**). DAPI stained nuclei in blue. Selected areas are magnified to the right. Underlined letters indicate residues substituted in Gαo-Nt^{7}. Pearson's co-localization analysis of GFP-fusions and mRFP-Lact-C2 (**e**). Box plots indicate median (middle line), 25th, 75th percentile (box), and lowest, highest value (whiskers); 3 independent experiments (Gαo-Nt^{7}, *n*=40; MGNC-Nt^{7}, *n*=39; GalT, *n*=42). **f**, Localization of MGNC-Nt^{7}-GFP upon 2-bromopalmitate (2-BrPal) incubation. **g**,**h**, Expression of GFP-fusions for full-length Gαo (Gαo-GFP), its MGNC mutant (MGNC-Gαo-GFP), or Gαo carrying the MGSLCSR sequence (MGSLCSR-Gαo-GFP). Immunodetection as in (**a**). Quantification of the expression of GFP-fusions (**h**). Data as mean ± s.e.m.; 3 independent experiments. **i**,**j**, MGNC-Gαo-GFP localization after 2-BrPal incubation (**i**) and MGSLCSR-Gαo-GFP under normal culture conditions (**j**). **k**, HeLa cells expressing Gαo-Nt^{7}-GFP (top), MGNC-Nt^{7}-GFP (middle), and MGSLCSR-Nt^{7}-GFP (bottom) were immunostained against GM130. Boxed areas are enlarged to the right. **l**-**o**, Live imaging of N2a cells co-expressing Gαo-Nt^{7}-GFP (**l**) or MGNC-Nt^{7}-GFP (**m**) and MannII-mRFP (bottom right insets). Representative images of the Nt^{7}-GFP constructs at the time of DMSO (Control) addition (0 min) and after 45 min. Quantification of PM content of Gαo-Nt^{7} (**n**) and MGNC-Nt^{7} (**o**) from Control and Palmostatin B (PalmB) treated cells in 5 min intervals and starting at t=0. Data represent mean ± s.d. (Gαo-Nt^{7} control, *n*=20; Gαo-Nt^{7} PalmB, *n*=20; MGNC-Nt^{7} control, *n*=20; MGNC-Nt^{7} PalmB, *n*=20). **p**,**q**, Localization of Gαo-GFP (**p**) and MGNC-Gαo-GFP (**q**) in N2a cells after 45 min of PalmB treatment. **c**,**d**,**f**,**i**-**m**,**p**,**q**, Scale bars, 10 µm. *P* values were determined using one-sample *t*-test for **b**,**h** and one-way ANOVA Tukey test for **e**. ns: not significant. Source data are provided as a Source Data file.

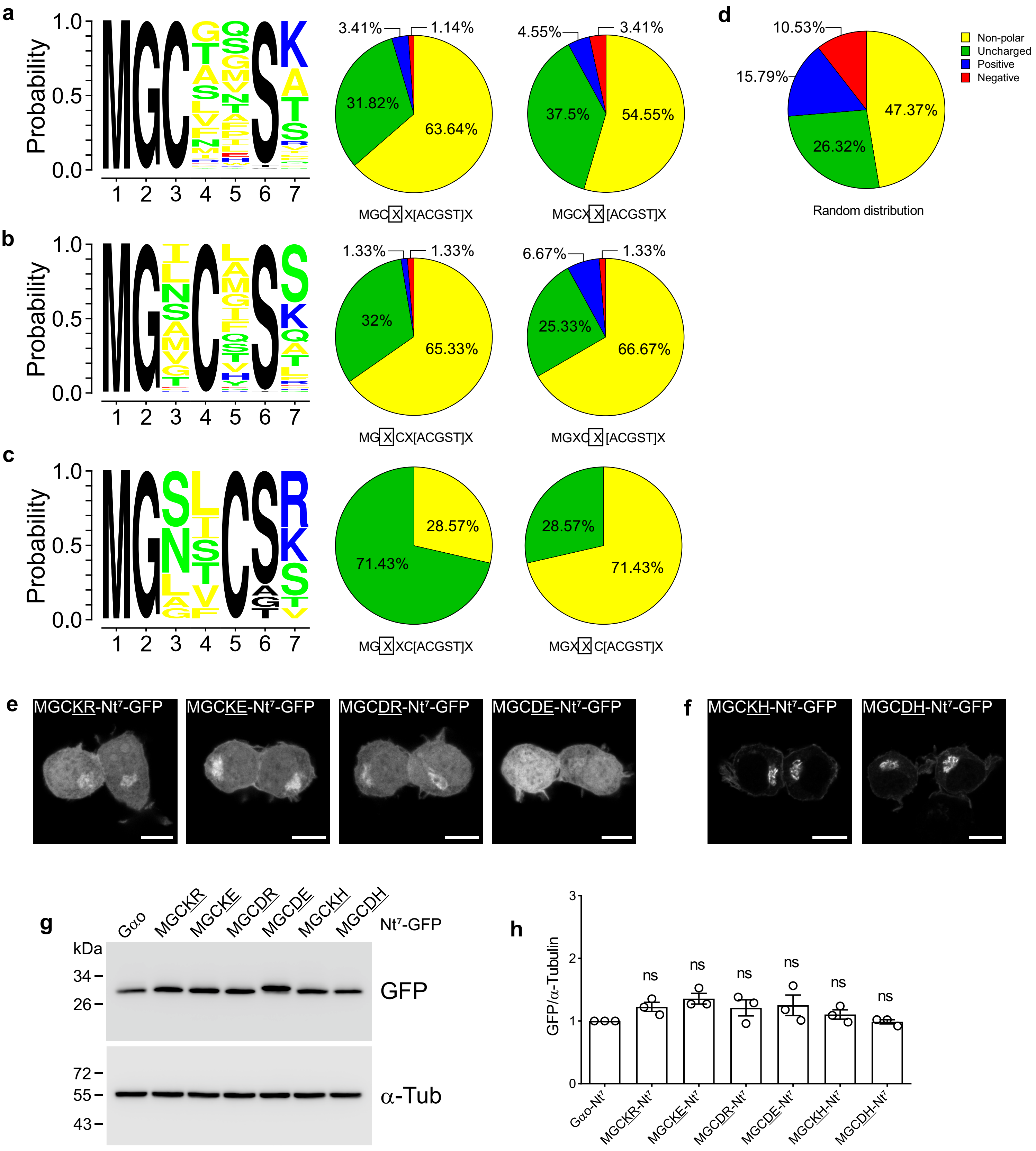

a
b
c
d
Probability
MGCXX[ACGST]X
MGCXX[ACGST]X
MGXCX[ACGST]X
MGXXC[ACGST]X
Random distribution
Non-polar
Uncharged
Positive
Negative
e
MGCKR-Nt7-GFP
MGCKE-Nt7-GFP
MGCDR-Nt7-GFP
MGCDE-Nt7-GFP
f
MGCKH-Nt7-GFP
MGCDH-Nt7-GFP
g
Gαo
MGCKR
MGCKE
MGCDR
MGCDE
MGCKH
MGCDH
Nt7-GFP
kDa
GFP
α-Tub
h
GFP/α-Tubulin
ns
Gαo-Nt7
MGCKR-Nt7
MGCKE-Nt7
MGCDR-Nt7
MGCDE-Nt7
MGCKH-Nt7
MGCDH-Nt7

Supplementary Figure 3. Eukaryotic Gα-Nt7 sequences

a-**d**, Logo representations (left) and quantification of frequency of amino acid polarity groups (right) of the unique Gα-Nt^7 sequences found in eukaryotic genomes of the categories Cys3 (**a**), Cys4 (**b**) and Cys5 (**c**). Frequency of a random distribution of amino acids (**d**). Amino acid polarity groups are color-coded as indicated in (**d**). **e**,**f**, Effect of positive and negative residues in the membrane association of Gαo-Nt^7-GFP mutants inferred from their localization in N2a cells. Positions 4 and 5 (underlined) in Gαo-Nt^7 were point mutated to introduce negative (Asp or Glu) or positive (Arg, Lys or His) residues as indicated in the figures. Scale bars, 10 µm. **g**,**h**, Western blot analysis of N2a cells transfected as in **e** and **f**. Antibody against GFP was used to detect the expression level of Nt^7-GFP constructs and α-Tubulin (α-Tub) as loading control (**g**). Quantification revealed no significant variation among the constructs (**h**). Data shown as the mean ± s.e.m. from 3 independent experiments. *P* values were determined using one-sample *t*-test for **h**. ns: not significant. Source data are provided as a Source Data file.

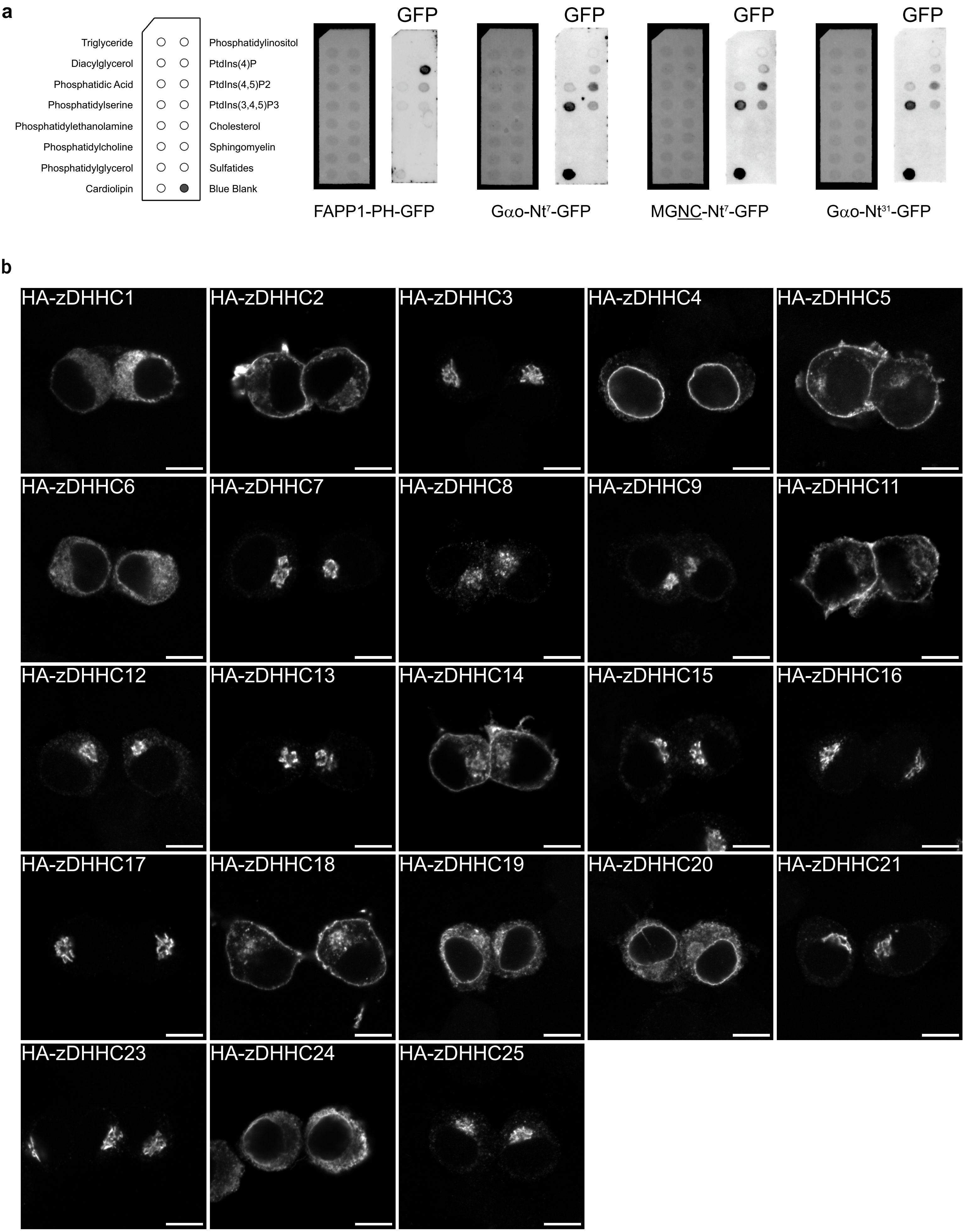
a
Triglyceride
Diacylglycerol
Phosphatidic Acid
Phosphatidylserine
Phosphatidylethanolamine
Phosphatidylcholine
Phosphatidylglycerol
Cardiolipin
Phosphatidylinositol
PtdIns(4)P
PtdIns(4,5)P2
PtdIns(3,4,5)P3
Cholesterol
Sphingomyelin
Sulfatides
Blue Blank
GFP
GFP
GFP
GFP
FAPP1-PH-GFP
Gαo-Nt7-GFP
MGNC-Nt7-GFP
Gαo-Nt31-GFP
b
HA-zDHHC1
HA-zDHHC2
HA-zDHHC3
HA-zDHHC4
HA-zDHHC5
HA-zDHHC6
HA-zDHHC7
HA-zDHHC8
HA-zDHHC9
HA-zDHHC11
HA-zDHHC12
HA-zDHHC13
HA-zDHHC14
HA-zDHHC15
HA-zDHHC16
HA-zDHHC17
HA-zDHHC18
HA-zDHHC19
HA-zDHHC20
HA-zDHHC21
HA-zDHHC23
HA-zDHHC24
HA-zDHHC25

Supplementary Figure 4. Subcellular localization of zDHHCs in N2a cells

a, A protein-lipid overlay assay on membrane strips spotted with different lipids as indicated in the illustration (left). Membrane strips were incubated with extracts from N2a cells expressing the control FAPP1-PH-GFP, Gαo-Nt^{7}-GFP, MGNC-Nt^{7}-GFP or Gαo-Nt^{31}-GFP, and detection of the constructs was done using an Ab against GFP. Images of the membrane strips are shown side-by-side with the corresponding Ab signals. **b**, N2a cells expressing the HA-tagged zDHHC collection were immunostained against HA and GM130 (not shown) to determine the subcellular localization of zDHHCs. Scale bars, 10 µm.

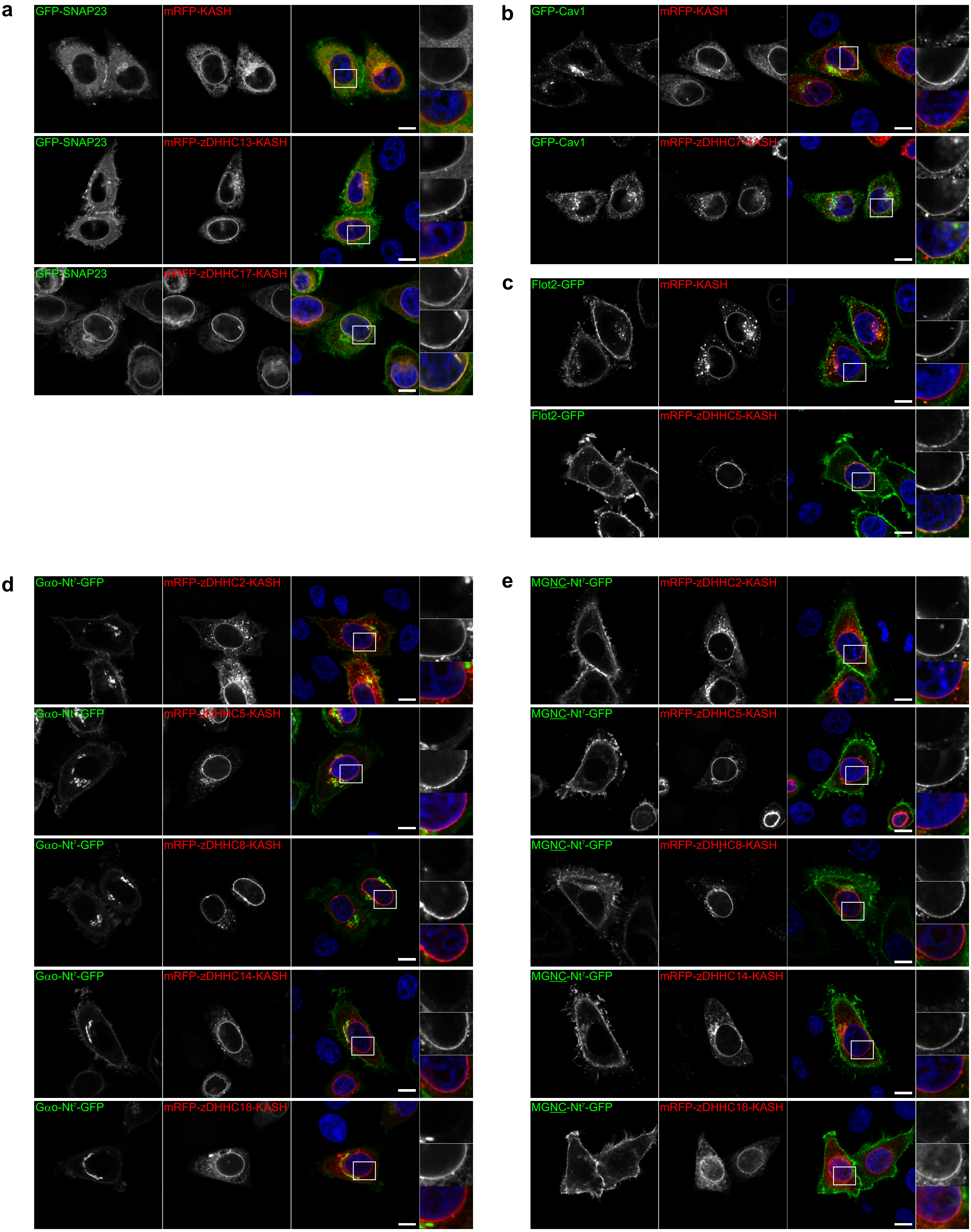

a
GFP-SNAP23
mRFP-KASH
GFP-SNAP23
mRFP-zDHHC13-KASH
GFP-SNAP23
mRFP-zDHHC17-KASH
b
GFP-Cav1
mRFP-KASH
GFP-Cav1
mRFP-zDHHC7-KASH
c
Flot2-GFP
mRFP-KASH
Flot2-GFP
mRFP-zDHHC5-KASH
d
Gαo-Nt^7-GFP
mRFP-zDHHC2-KASH
Gαo-Nt^7-GFP
mRFP-zDHHC5-KASH
Gαo-Nt^7-GFP
mRFP-zDHHC8-KASH
Gαo-Nt^7-GFP
mRFP-zDHHC14-KASH
Gαo-Nt^7-GFP
mRFP-zDHHC18-KASH
e
MGNC-Nt^7-GFP
mRFP-zDHHC2-KASH
MGNC-Nt^7-GFP
mRFP-zDHHC5-KASH
MGNC-Nt^7-GFP
mRFP-zDHHC8-KASH
MGNC-Nt^7-GFP
mRFP-zDHHC14-KASH
MGNC-Nt^7-GFP
mRFP-zDHHC18-KASH

Supplementary Figure 5. Validation of the S-palmitoylation at the ONM – SwissKASH – assay

a-**c**, The SwissKASH assay in HeLa cells applied to GFP-fusions of SNAP23 (GFP-SNAP23; **a**), caveolin-1 (GFP-Cav1; **b**), and flotllin-2 (Flot2-GFP; **c**). GFP-SNAP23 was efficiently recruited to the ONM by the KASH-constructs of zDHHC13 (mRFP-zDHHC13-KASH) and zDHHC17 (mRFP-zDHHC17-KASH), but not by the control mRFP-KASH (**a**). Similarly, GFP-Cav1 targeted the ONM by the co-expression of mRFP-zDHHC7-KASH (**b**), and Flot2-GFP by mRFP-zDHHC5-KASH (**c**), but not by the control mRFP-KASH (**b**,**c**). **d**,**e**, The SwissKASH assay for Gαo-Nt^{7}-GFP (**d**) and MGNC-Nt^{7}-GFP (**e**) using the mRFP- and KASH-fusions of the PM-associated zDHHC2, 5, 8, 14, and 18. Underlined letters indicate residues substituted in Gαo-Nt^{7}. **a**-**e**, DAPI staining of nuclei in blue, boxed areas are enlarged to the right. Scale bars, 10 µm.

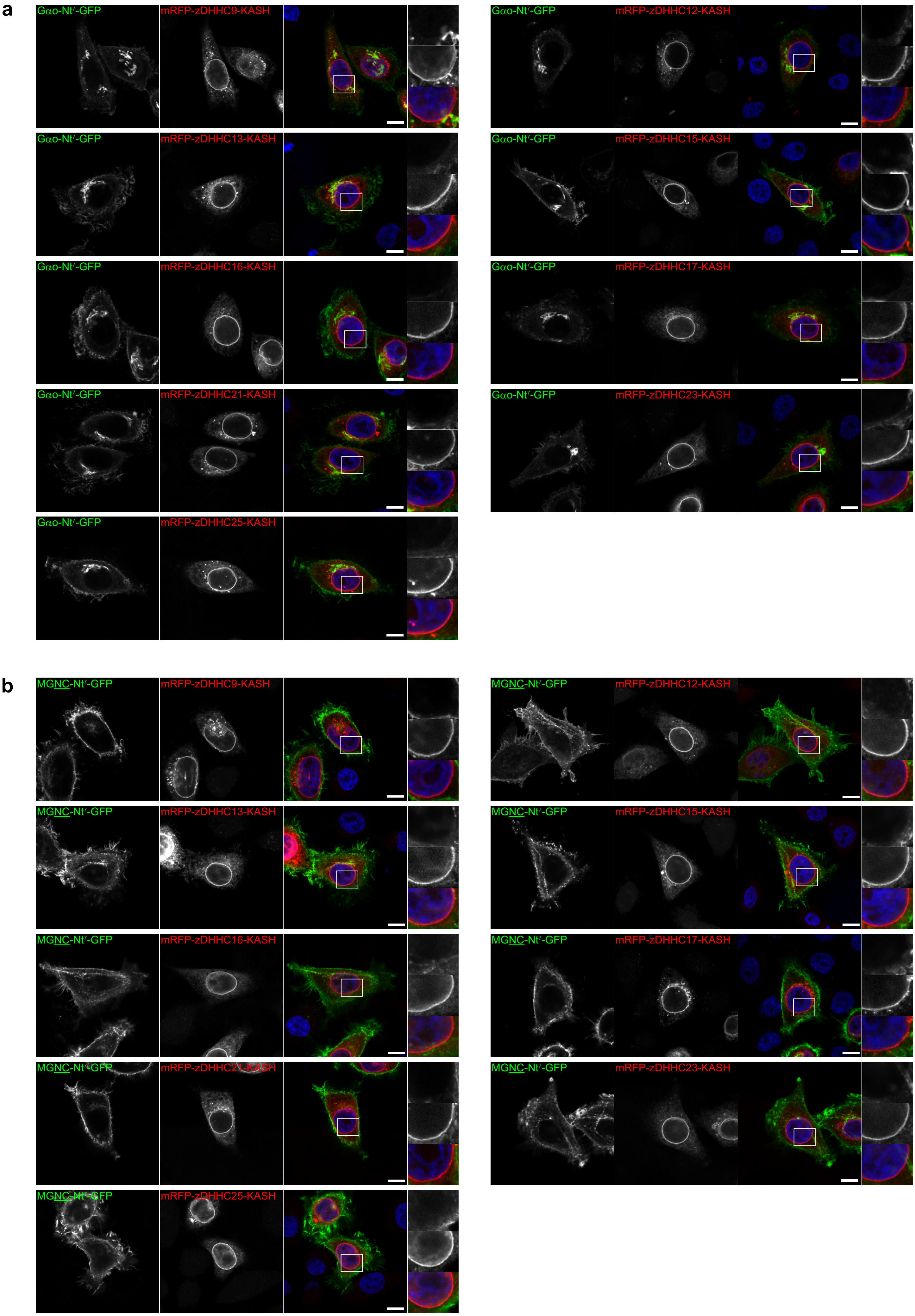

a
Gαo-Nt^7-GFP
mRFP-zDHHC9-KASH
mRFP-zDHHC12-KASH
mRFP-zDHHC13-KASH
mRFP-zDHHC15-KASH
mRFP-zDHHC16-KASH
mRFP-zDHHC17-KASH
mRFP-zDHHC21-KASH
mRFP-zDHHC23-KASH
mRFP-zDHHC25-KASH
b
MGNC-Nt^7-GFP

Supplementary Figure 6. Golgi-localized zDHHCs

a,**b**, The SwissKASH assay in HeLa cells applied to Gαo-Nt^7-GFP (**a**) and MGNC-Nt^7-GFP (**b**) using the mRFP- and KASH-fusions of the Golgi-associated zDHHC9, 12, 13, 15, 16, 17, 21, 23 and 25. Underlined letters indicate residues substituted in Gαo-Nt^7. A blue DAPI staining marks nuclei. Selected areas are zoomed-in to the right. Scale bars, 10 µm.

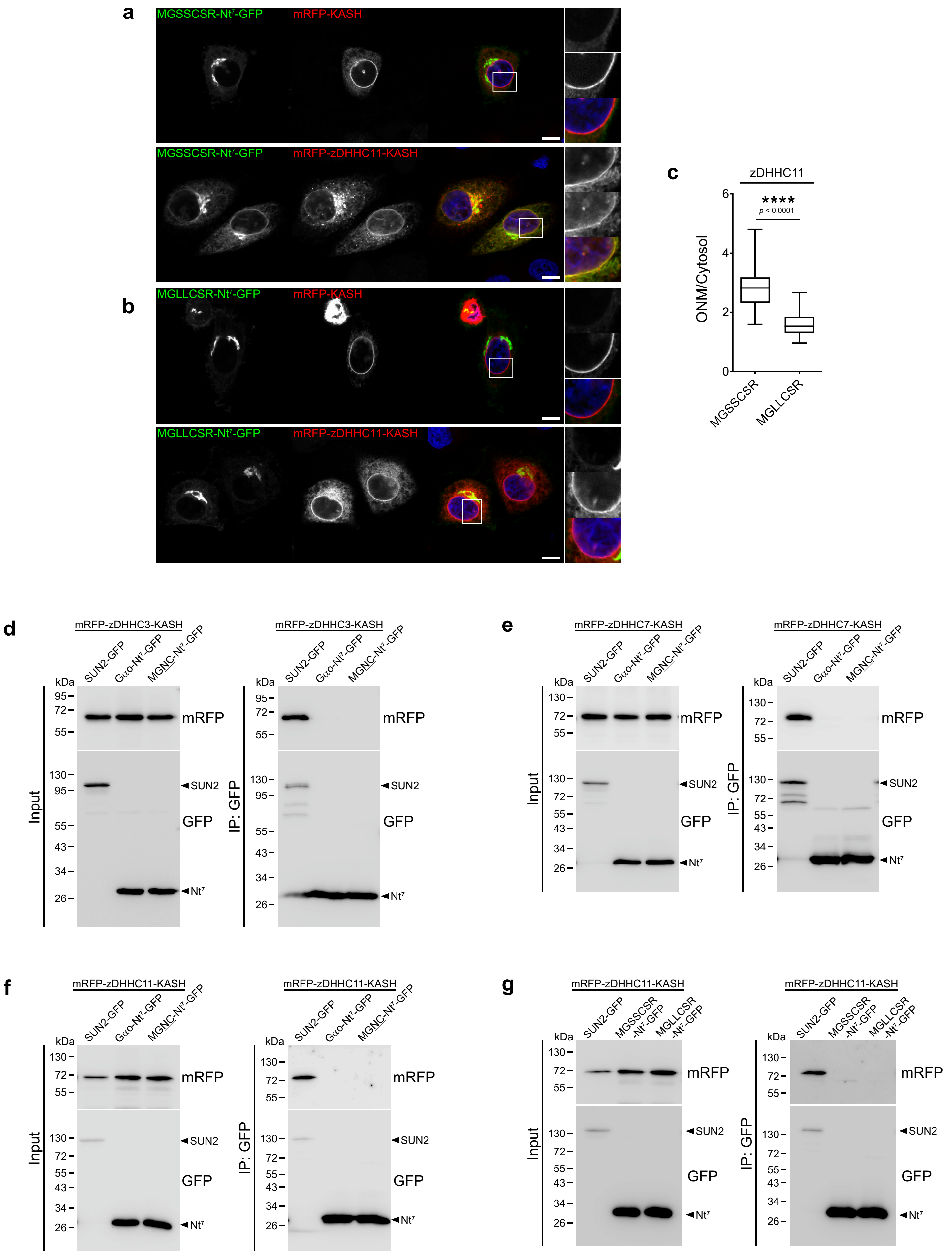
a
MGSSCSR-Nt7-GFP
mRFP-KASH
mRFP-zDHHC11-KASH
b
MGLLCSR-Nt7-GFP
mRFP-KASH
mRFP-zDHHC11-KASH
c
zDHHC11
****
p < 0.0001
ONM/Cytosol
0
2
4
6
MGSSCSR
MGLLCSR
d
mRFP-zDHHC3-KASH
SUN2-GFP
Gαo-Nt7-GFP
MGNC-Nt7-GFP
kDa
Input
IP: GFP
mRFP
SUN2
GFP
Nt7
e
mRFP-zDHHC7-KASH
f
mRFP-zDHHC11-KASH
g
MGSSCSR-Nt7-GFP
MGLLCSR-Nt7-GFP
130
95
72
55
43
34
26

Supplementary Figure 7. Substrate specificity of zDHHC11

a-**c**, The SwissKASH assay in HeLa cells was applied to MGSSCSR-Nt^7-GFP (**a**) and MGLLCSR-Nt^7-GFP (**b**) using the control mRFP-KASH or mRFP-zDHHC11-KASH constructs. Marked areas are magnified to the right. Quantification of mean fluorescence intensity ratio of GFP-constructs at the ONM versus cytosol (**c**). Box plots indicate median (middle line), 25th, 75th percentile (box), and lowest, highest value (whiskers) from 3 independent experiments (MGSSCSR-Nt^7, *n*=49; MGLLCSR-Nt^7, *n*=49). **d**-**g**, Immunoprecipitation (IP) of SUN2-GFP, Gαo-Nt^7-GFP and MGNC-Nt^7-GFP (**d**-**f**) or MGSSCSR-Nt^7-GFP and MGLLCSR-Nt^7-GFP (**g**) under the co-expression of mRFP-zDHHC3-KASH (**d**), mRFP-zDHHC7-KASH (**e**) or mRFP-zDHHC11-KASH (**f**,**g**). Underlined letters indicate residues substituted in Gαo-Nt^7. Note that only SUN2-GFP co-precipitated all mRFP-zDHHC-KASH constructs. Antibodies against GFP and mRFP were used for detection. **a**,**b**, Scale bars, 10 µm. *P* values were determined using two-sided unpaired *t*-test for **c**. Source data are provided as a Source Data file.

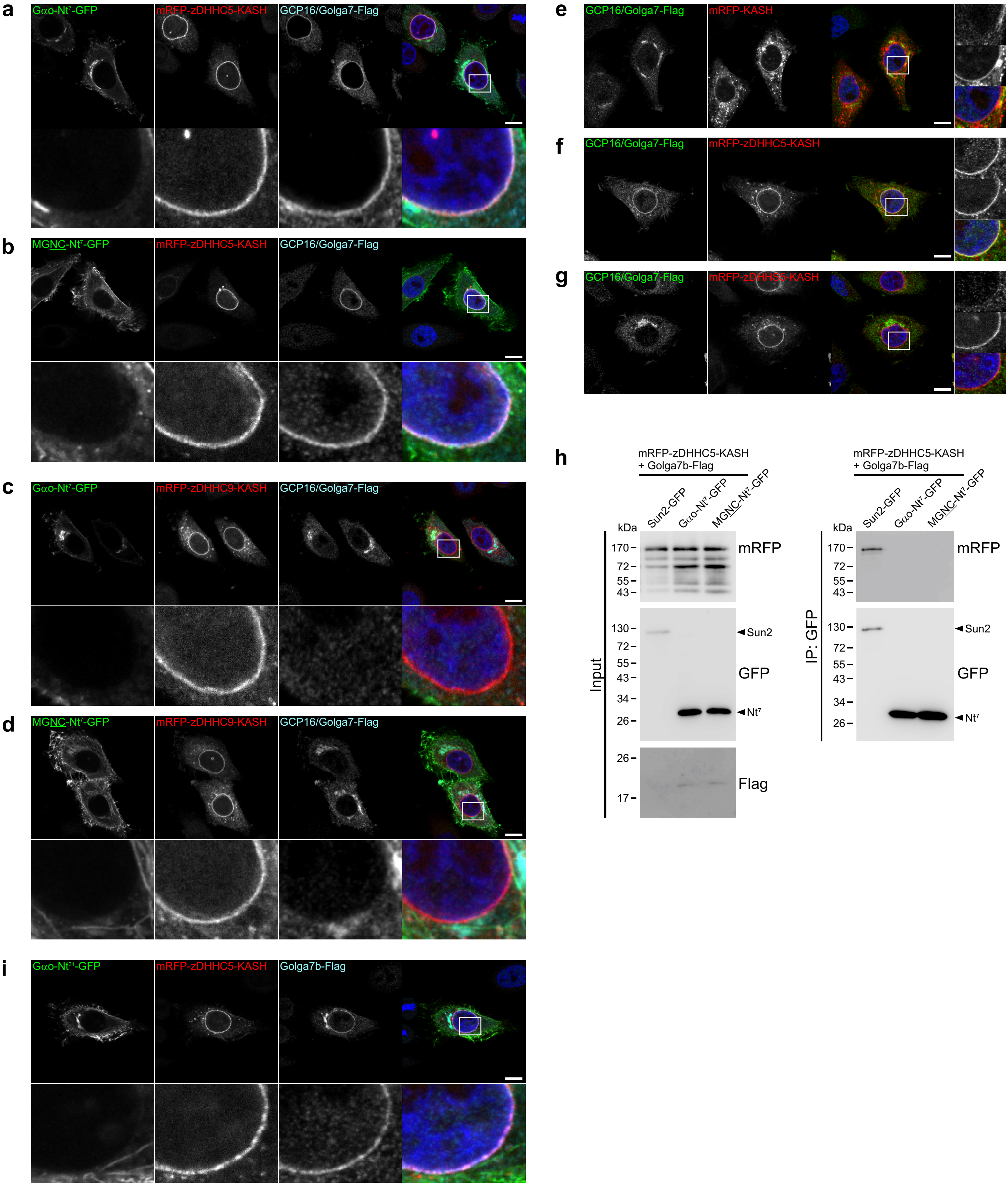
a
Gαo-Nt7-GFP
mRFP-zDHHC5-KASH
GCP16/Golga7-Flag
b
MGNC-Nt7-GFP
mRFP-zDHHC5-KASH
GCP16/Golga7-Flag
c
Gαo-Nt7-GFP
mRFP-zDHHC9-KASH
GCP16/Golga7-Flag
d
MGNC-Nt7-GFP
mRFP-zDHHC9-KASH
GCP16/Golga7-Flag
i
Gαo-Nt31-GFP
mRFP-zDHHC5-KASH
Golga7b-Flag
e
GCP16/Golga7-Flag
mRFP-KASH
f
GCP16/Golga7-Flag
mRFP-zDHHC5-KASH
g
GCP16/Golga7-Flag
mRFP-zDHHS5-KASH
h
mRFP-zDHHC5-KASH
+ Golga7b-Flag
Sun2-GFP
Gαo-Nt7-GFP
MGNC-Nt7-GFP
kDa
170
72
55
43
mRFP
130
72
55
43
34
26
Sun2
GFP
Nt7
26
17
Flag
Input
IP: GFP

Supplementary Figure 8. Accessory proteins in the SwissKASH assay

a-**d**, The SwissKASH assay for Gαo-Nt7-GFP (**a**,**c**) and MGNC-Nt7-GFP (**b**,**d**) using mRFP-zDHHC5-KASH (**a**,**b**) or mRFP-zDHHC9-KASH (**c**,**d**), both in conjunction with GCP16/Golga7-Flag. Cells were immunostained against Flag-tag and nuclei were stained in blue with DAPI. Marked regions are magnified at the bottom panels. Underlined letters indicate residues substituted in Gαo-Nt^7. **e**-**g**, The SwissKASH assay applied for GCP16/Golga7-Flag using the control mRFP-KASH (**e**), mRFP-zDHHC5-KASH (**f**) or its DHHS-inactive mutant (mRFP-zDHHS5-KASH; **g**). Note that the strong ONM accumulation of GCP16/Golga7-Flag induced by the KASH-fusion of zDHHC5 is not seen by its DHHS mutant. Cells were immunostained against Flag-tag and DAPI stained nuclei in blue. Selected areas are enlarged to the right. **h**, Immunoprecipitation (IP) of SUN2-GFP, Gαo-Nt^7-GFP and MGNC-Nt^7-GFP by the co-expression of mRFP-zDHHC5-KASH and Golga7b-Flag. Note that only SUN2-GFP co-precipitated mRFP-zDHHC5-KASH. Golga7b was weakly detected in the input and not detected at all in the IP (no shown). Antibodies (Abs) against GFP, mRFP, and Flag-tag were used for detection. **i**, The SwissKASH assay in HeLa cells was applied to Gαo-Nt^{31}-GFP using mRFP-zDHHC5-KASH together with Golga7b-Flag. Ab against Flag-tag was used for immunostaining and DAPI for staining nuclei in blue. A marked area is magnified at the bottom. **a**-**g**,**i**, Scale bars, 10 µm. Source data are provided as a Source Data file.

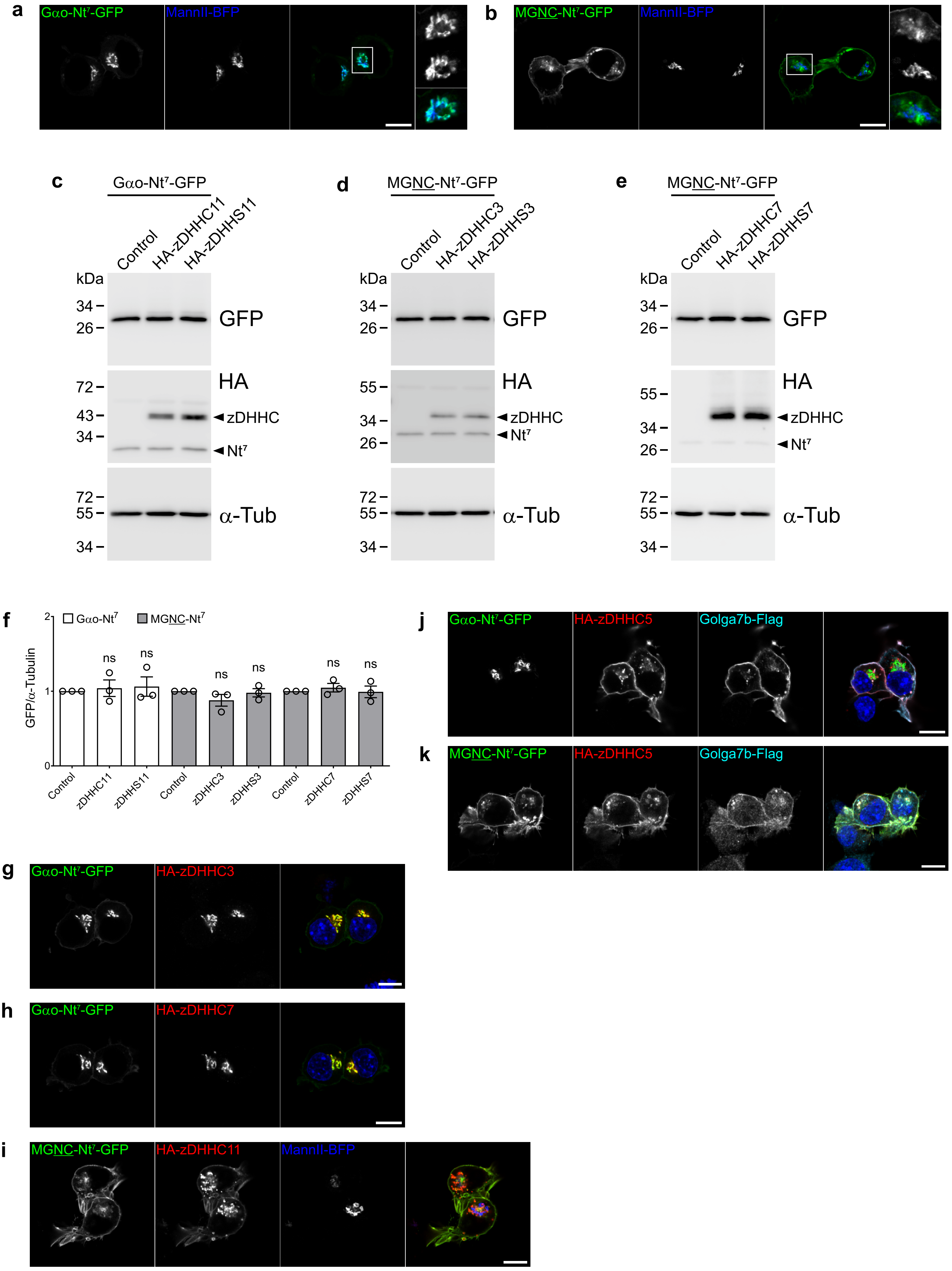
a
Gαo-Nt⁷-GFP
MannII-BFP
b
MGNC-Nt⁷-GFP
MannII-BFP
c
Gαo-Nt⁷-GFP
Control
HA-zDHHC11
HA-zDHHS11
kDa
34
26
GFP
72
43
34
HA
◄zDHHC
◄Nt⁷
72
55
34
α-Tub
d
MGNC-Nt⁷-GFP
Control
HA-zDHHC3
HA-zDHHS3
kDa
34
26
GFP
55
34
26
HA
◄zDHHC
◄Nt⁷
72
55
34
α-Tub
e
MGNC-Nt⁷-GFP
Control
HA-zDHHC7
HA-zDHHS7
kDa
34
26
GFP
55
34
26
HA
◄zDHHC
◄Nt⁷
72
55
34
α-Tub
f
Gαo-Nt⁷
MGNC-Nt⁷
GFP/α-Tubulin
2
1
0
ns
ns
ns
ns
ns
ns
Control
zDHHC11
zDHHS11
Control
zDHHC3
zDHHS3
Control
zDHHC7
zDHHS7
g
Gαo-Nt⁷-GFP
HA-zDHHC3
h
Gαo-Nt⁷-GFP
HA-zDHHC7
i
MGNC-Nt⁷-GFP
HA-zDHHC11
MannII-BFP
j
Gαo-Nt⁷-GFP
HA-zDHHC5
Golga7b-Flag
k
MGNC-Nt⁷-GFP
HA-zDHHC5
Golga7b-Flag

Supplementary Figure 9. Localization and expression of Nt[7] constructs upon co-expression of zDHHCs

a,**b**, Representative images of N2a cells expressing the Golgi marker MannII-BFP together with Gαo-Nt[7]-GFP (**a**) or MGNC-Nt[7]-GFP (**b**). Selected areas are zoomed-in to the right. Underlined letters indicate residues substituted in Gαo-Nt[7]. **c**-**f**, Western blot (WB) analysis of N2a cells. Cells were co-transfected with Gαo-Nt[7]-GFP together with empty pcDNA3.1(+) plasmid (Control), HA-tagged zDHHC11 (HA-zDHHC11) or its DHHS-inactive mutant (HA-zDHHS11) (**c**). MGNC-Nt[7]-GFP was co-transfected with control plasmid, HA-zDHHC3 or HA-zDHHS3 (**d**), as well as with HA-zDHHC7 or HA-zDHHS7 (**e**). Antibody (Ab) against GFP was used to detect the expression level of Nt[7] constructs, anti-HA detected HA-zDHHCs and α-Tubulin (α-Tub) served as loading control (**c**-**e**). Arrowheads marked as Nt[7] point to anti-GFP residual signals. Quantification showed no significant (ns) difference between the Nt[7]-GFP constructs (**f**). Data shown as the mean ± s.e.m. from 3 independent experiments. **g**-**i**, Confocal images of N2a cells expressing Gαo-Nt[7]-GFP (**g**,**h**) or MGNC-Nt[7]-GFP (**i**) together with HA-zDHHC3 (**g**), HA-zDHHC7 (**h**), or HA-zDHHC11 with MannII-BFP (**i**). Cells were immunostained against the HA-tag. DAPI staining was used to visualize nuclei (**g**,**h**). **j**,**k**, Co-expression of Gαo-Nt[7]-GFP (**j**) or MGNC-Nt[7]-GFP (**k**) with HA-zDHHC5 and Golga7b-Flag in N2a cells. Immunostainings against the HA- and Flag-tags were used for detection and DAPI staining to mark nuclei in blue. **a**,**b**,**g**-**k**, Scale bars, 10 µm. *P* values were determined using one-sample *t*-test for **f**. ns: not significant. Source data are provided as a Source Data file.

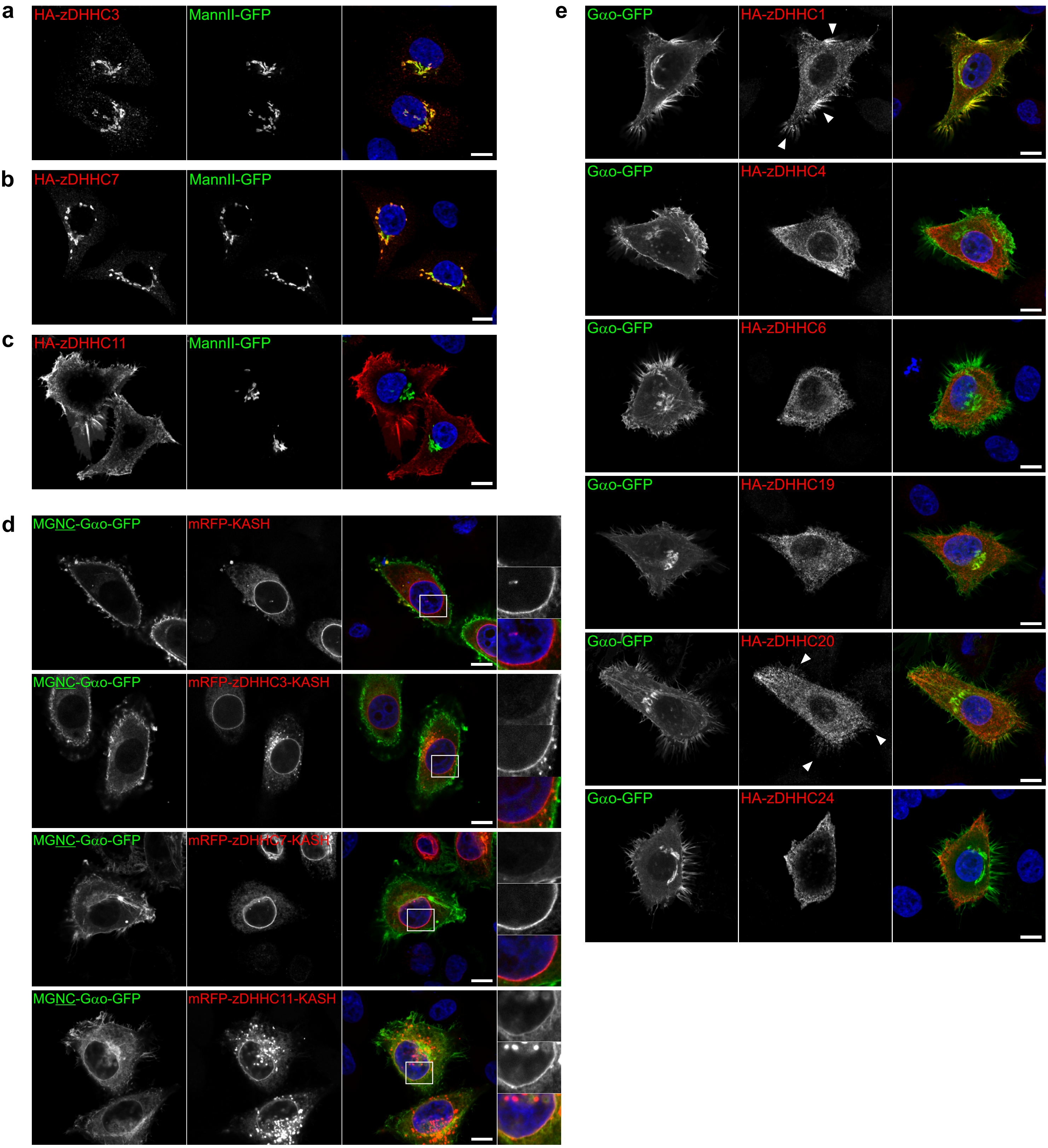

a
HA-zDHHC3
MannII-GFP
b
HA-zDHHC7
MannII-GFP
c
HA-zDHHC11
MannII-GFP
d
MGNC-Gαo-GFP
mRFP-KASH
MGNC-Gαo-GFP
mRFP-zDHHC3-KASH
MGNC-Gαo-GFP
mRFP-zDHHC7-KASH
MGNC-Gαo-GFP
mRFP-zDHHC11-KASH
e
Gαo-GFP
HA-zDHHC1
Gαo-GFP
HA-zDHHC4
Gαo-GFP
HA-zDHHC6
Gαo-GFP
HA-zDHHC19
Gαo-GFP
HA-zDHHC20
Gαo-GFP
HA-zDHHC24

Supplementary Figure 10. Full-length Gαo and the ER-localized zDHHCs

a-**c**, Confocal images of HeLa cells co-expressing HA-zDHHC3 (**a**), HA-zDHHC7 (**b**), or HA-DHHC11 (**c**) with the Golgi marker MannII-GFP. Cells were immunostained against the HA-tag, and DAPI stained in blue the nuclei. **d**, The SwissKASH assay for the MGNC mutant of full-length Gαo (MGNC-Gαo-GFP) using the mRFP-KASH control, zDHHC3, 7 and 11. Note that MGNC-Gαo-GFP efficiently targeted the ONM by co-expression of zDHHC11, but not of zDHHC3 and 7. Underlined letters indicate residues substituted in Gαo. DAPI staining of nuclei in blue. Selected regions are zoomed-in to the right. **e**, Representative images of HeLa cells co-expressing the GFP-fusion of the full-length Gαo (Gαo-GFP) together with the HA-tagged zDHHCs that localize at the ER (HA-zDHHC1, 4, 6, 19, 20 and 24). Cells were immunostained against the HA-tag and nuclei were stained in blue with DAPI. White arrowheads point to zDHHC1 and 20 at filopodia, indicating their localization at the PM. Note that co-expression of the ER-localized zDHHCs seemed not to affect the overall localization of Gαo-GFP. **a**-**e**, Scale bars, 10 µm.

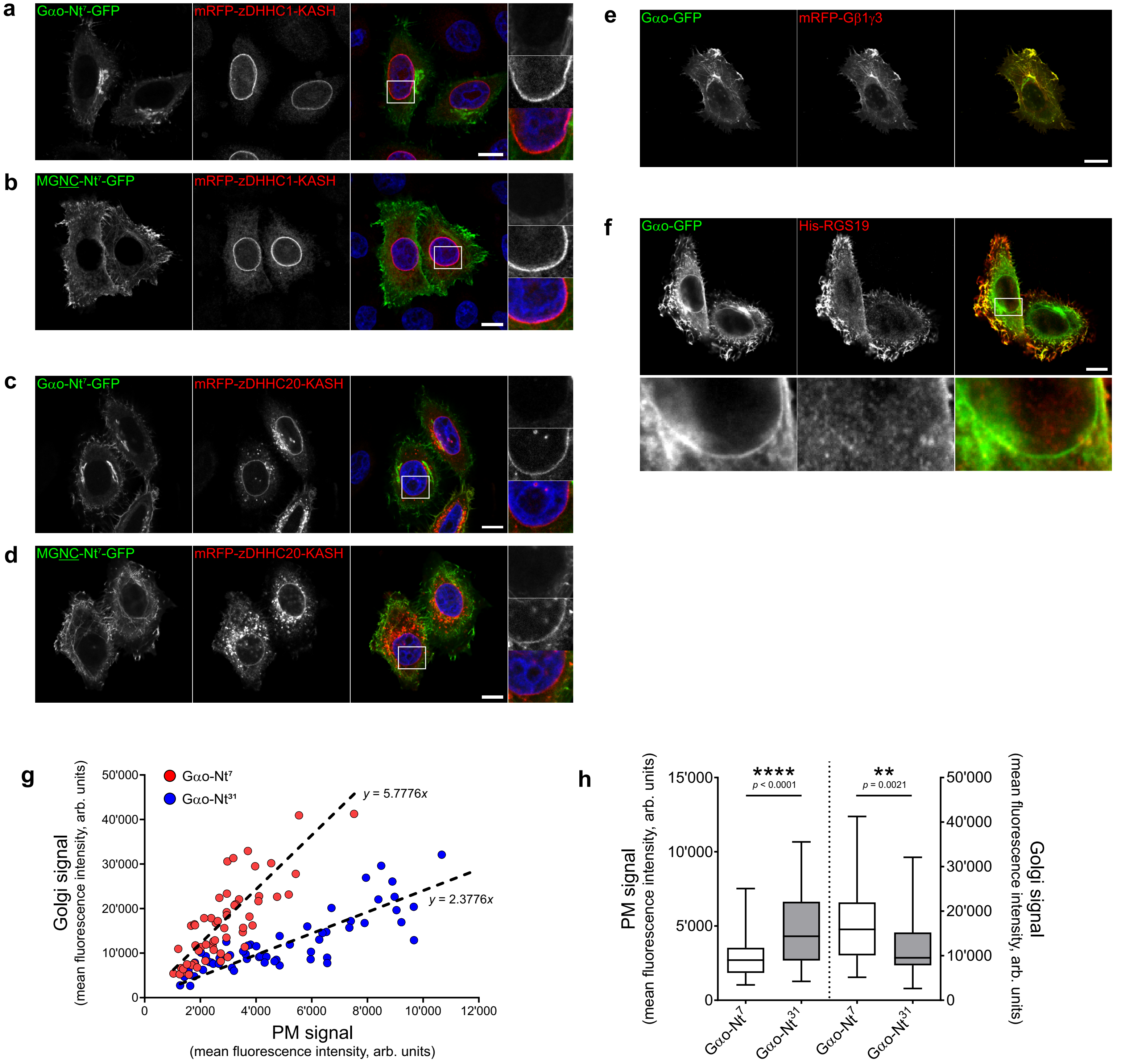
a
Gαo-Nt7-GFP
mRFP-zDHHC1-KASH
b
MGNC-Nt7-GFP
mRFP-zDHHC1-KASH
c
Gαo-Nt7-GFP
mRFP-zDHHC20-KASH
d
MGNC-Nt7-GFP
mRFP-zDHHC20-KASH
e
Gαo-GFP
mRFP-Gβ1γ3
f
Gαo-GFP
His-RGS19
g
Gαo-Nt7
Gαo-Nt31
y = 5.7776x
y = 2.3776x
Golgi signal
(mean fluorescence intensity, arb. units)
PM signal
(mean fluorescence intensity, arb. units)
0
10'000
20'000
30'000
40'000
50'000
0
2'000
4'000
6'000
8'000
10'000
12'000
h
****
p < 0.0001
**
p = 0.0021
PM signal
(mean fluorescence intensity, arb. units)
Golgi signal
(mean fluorescence intensity, arb. units)
0
5'000
10'000
15'000
0
10'000
20'000
30'000
40'000
50'000
Gαo-Nt7
Gαo-Nt31
Gαo-Nt7
Gαo-Nt31

Supplementary Figure 11. The SwissKASH assay carrying zDHHC1 and 20

a-**d**, The SwissKASH assay was applied to Gαo-Nt^7-GFP (**a**,**c**) and MGNC-Nt^7-GFP (**b**,**d**) using mRFP-zDHHC1-KASH (**a**,**b**) or mRFP-zDHHC20-KASH (**c**,**d**). Underlined letters indicate residues substituted in Gαo-Nt^7. A blue DAPI staining marks nuclei. Marked regions are magnified to the right. **e**, Localization of full-length Gαo-GFP and mRFP-Gβ1γ3 in HeLa cells co-expressing the SwissKASH control construct BFP-KASH (not shown). **f**, The SwissKASH assay applied to Gαo-GFP and the His_6-tagged RGS19 (His-RGS19) using BFP-zDHHC11-KASH (not shown). Cells were immunostained against the His_6-tag. A selected area is enlarged at the bottom. **g**,**h**, Quantification of the apparent PM and Golgi localization of Gαo-Nt^7-GFP and Gαo-Nt^{31}-GFP from images recorded using the same confocal setting. A scatter plot (**g**) shows that to a similar Golgi content of both constructs, Gαo-Nt^{31} showed higher PM values (arbitrary (arb.) units). Dashed lines represent linear regressions with their equations alongside. A bar graph (**h**) of the pooled data illustrates the variation between the relative Golgi and PM content of both constructs. Box plots indicate median (middle line), 25th, 75th percentile (box), and lowest, highest value (whiskers) from 2 independent experiments (Gαo-Nt^7, n=56; Gαo-Nt^{31}, n=57). **a**-**f**, Scale bars, 10 µm. P values were determined using two-sided unpaired t-test for **h**. Source data are provided as a Source Data file.

Supplementary References

1. Crooks, G.E., Hon, G., Chandonia, J.M. & Brenner, S.E. WebLogo: a sequence logo generator. *Genome Res* **14**, 1188-1190 (2004).
2. Castrec, B. *et al.* Structural and genomic decoding of human and plant myristoylomes reveals a definitive recognition pattern. *Nat Chem Biol* **14**, 671-679 (2018).
3. Garcia-Nafria, J., Nehme, R., Edwards, P.C. & Tate, C.G. Cryo-EM structure of the serotonin 5-HT1B receptor coupled to heterotrimeric Go. *Nature* **558**, 620-623 (2018).
4. Yuan, D. *et al.* Activation of the alpha2B adrenoceptor by the sedative sympatholytic dexmedetomidine. *Nat Chem Biol* **16**, 507-512 (2020).
5. Maeda, S., Qu, Q., Robertson, M.J., Skiniotis, G. & Kobilka, B.K. Structures of the M1 and M2 muscarinic acetylcholine receptor/G-protein complexes. *Science* **364**, 552-557 (2019).
6. Kaya, A.I. *et al.* A Conserved Hydrophobic Core in Galphai1 Regulates G Protein Activation and Release from Activated Receptor. *J Biol Chem* **291**, 19674-19686 (2016).
7. Wall, M.A. *et al.* The structure of the G protein heterotrimer Gi alpha 1 beta 1 gamma 2. *Cell* **83**, 1047-1058 (1995).
8. Koehl, A. *et al.* Structure of the micro-opioid receptor-Gi protein complex. *Nature* **558**, 547-552 (2018).
9. Hua, T. *et al.* Activation and Signaling Mechanism Revealed by Cannabinoid Receptor-Gi Complex Structures. *Cell* **180**, 655-665 e618 (2020).
10. Krishna Kumar, K. *et al.* Structure of a Signaling Cannabinoid Receptor 1-G Protein Complex. *Cell* **176**, 448-458 e412 (2019).
11. Kato, H.E. *et al.* Conformational transitions of a neurotensin receptor 1-Gi1 complex. *Nature* **572**, 80-85 (2019).
