# Supplementary material for: Local and substrate-specific S-palmitoylation determines subcellular localization of Gαo
Source: Nat Commun. 2022 Apr 19;13:2072. doi: 10.1038/s41467-022-29685-8 (PMC9018777; doi:10.1038/s41467-022-29685-8)
Supplement: Supplementary file 3 — Description of Additional Supplementary Files [file 41467_2022_29685_MOESM3_ESM.pdf]

Description of Additional Supplementary Files

File name: Supplementary Data 1

Description: Eukaryotic species and their Gα subunit sequences (N-terminal 10 amino acids) with the corresponding Entrez IDs. Phylogenetic clade classification was according to the NCBI Taxonomy browser.

File name: Supplementary Data 2

Description: The unique Gα-Nt^7 sequences (in fasta format) found in eukaryotic genomes of the categories Cys3, Cys4 and Cys5.

File name: Supplementary Data 3

Description: Sequences of oligonucleotides used in this study.

File name: Supplementary Movie 1

Description: N2a cells expressing Gαo-Nt^7-GFP and the Golgi marker MannII-mRFP were treated with 50 µM Palmostatin B, and immediately recorded at one image per 30 seconds for 45 min as described in Methods. The Movie at 10 frames per second shows several cells displaying the changes in localization of Gαo-Nt^7 upon time.

File name: Supplementary Movie 2

Description: N2a cells expressing MGNC-Nt^7-GFP and the Golgi marker MannII-mRFP were treated and recorded as in Supplementary Movie 1. The Movie shows a group of cells displaying the changes in MGNC-Nt^7 localization upon time.

File name: Supplementary Movie 3

Description: HeLa cells expressing Gαo-Nt^7-FM4-GFP and the Golgi marker MannII-mRFP were treated with D/D solubilizer, and immediately recorded at one image per 5 seconds for 10 min as described in Methods. The Movie at 12 frames per second shows two representative cells displaying the Golgi accumulation of Gαo-Nt^7-FM4-GFP upon time.

File name: Supplementary Movie 4

Description: HeLa cells expressing MGNC-Nt^7-FM4-GFP and MannII-mRFP were prepared and recorded as in Supplementary Movie 3. The Movie shows two representative cells showing a mainly PM targeting of MGNC-Nt^7-FM4-GFP.
