# Supplementary material for: Local and substrate-specific S-palmitoylation determines subcellular localization of Gαo
Source: Nat Commun. 2022 Apr 19;13:2072. doi: 10.1038/s41467-022-29685-8 (PMC9018777; doi:10.1038/s41467-022-29685-8)
Supplement: Supplementary file 11 — Reporting Summary [file 41467_2022_29685_MOESM11_ESM.pdf]

nature research

Corresponding author(s): Gonzalo P. Solis
Vladimir L. Katanaev

Last updated by author(s): Mar 3, 2022

Reporting Summary

Nature Research wishes to improve the reproducibility of the work that we publish. This form provides structure for consistency and transparency in reporting. For further information on Nature Research policies, see our Editorial Policies and the Editorial Policy Checklist.

Statistics

For all statistical analyses, confirm that the following items are present in the figure legend, table legend, main text, or Methods section.

n/a	Confirmed	
☐	☒	The exact sample size (*n*) for each experimental group/condition, given as a discrete number and unit of measurement
☐	☒	A statement on whether measurements were taken from distinct samples or whether the same sample was measured repeatedly
☐	☒	The statistical test(s) used AND whether they are one- or two-sided<br>*Only common tests should be described solely by name; describe more complex techniques in the Methods section.*
☒	☐	A description of all covariates tested
☐	☒	A description of any assumptions or corrections, such as tests of normality and adjustment for multiple comparisons
☐	☒	A full description of the statistical parameters including central tendency (e.g. means) or other basic estimates (e.g. regression coefficient) AND variation (e.g. standard deviation) or associated estimates of uncertainty (e.g. confidence intervals)
☒	☐	For null hypothesis testing, the test statistic (e.g. *F*, *t*, *r*) with confidence intervals, effect sizes, degrees of freedom and *P* value noted<br>*Give P values as exact values whenever suitable.*
☒	☐	For Bayesian analysis, information on the choice of priors and Markov chain Monte Carlo settings
☒	☐	For hierarchical and complex designs, identification of the appropriate level for tests and full reporting of outcomes
☒	☐	Estimates of effect sizes (e.g. Cohen's *d*, Pearson's *r*), indicating how they were calculated

Our web collection on statistics for biologists contains articles on many of the points above.

Software and code

Policy information about availability of computer code

Data collection	Software: ZEN 2.3 (Zeiss), VisiView v4.00.10 (Visitron Systems), EvolutionCapt v18.11 (Vilber), ImageQuant TL v8.1.0.0 (Amersham)
Data analysis	Prism 9 (GraphPad) was used for statistic analysis and generation of graph images<br>ImageJ v1.53c was used for quantification of Western blot bands and mean fluorescence intensity of cell images, calculation of Pearson's correlation, and generation and edition of supplementary movies<br>EvolutionCapt v18.11 (Vilber) was used for edition of Western blot images<br>ZEN lite 3.3 (Zeiss) was used for edition of confocal images<br>ImageQuant TL v8.1.0.0 (Amersham) was used for quantification of images from metabolic radiolabeling with [3H]palmitate<br>WebLogo (weblogo.berkeley.edu) was used for generation of Logo representations<br>Inkscape v1.0.2 (inkscape.org) was used for the edition of Logo images and generation of vector graphics<br>CorelDRAW 2020 (Corel) was used for generation of figures<br>PyMOL v2.3 (pymol.org) was used for structure alignments

For manuscripts utilizing custom algorithms or software that are central to the research but not yet described in published literature, software must be made available to editors and reviewers. We strongly encourage code deposition in a community repository (e.g. GitHub). See the Nature Research guidelines for submitting code & software for further information.

Data

Policy information about availability of data

All manuscripts must include a data availability statement. This statement should provide the following information, where applicable:

- Accession codes, unique identifiers, or web links for publicly available datasets
- A list of figures that have associated raw data
- A description of any restrictions on data availability

Web links of RCSB-datasets for Gαo and Gαi1 structures used in this study: 6g79 (rcsb.org/structure/6G79), 6oik (rcsb.org/structure/6OIK), 6k41 (rcsb.org/structure/6K41), 1gg2 (rcsb.org/structure/1GG2), 5kdo (rcsb.org/structure/5KDO), 6ddf (rcsb.org/structure/6DDF), 6osa (rcsb.org/structure/6OSA), 6n4b (rcsb.org/structure/6N4B), 6kpf (rcsb.org/structure/6KPF), and 6k42 (rcsb.org/structure/6K42). The data that support the findings of this study are provided as Source Data, Supplementary Information, and Supplementary Data files.

Field-specific reporting

Please select the one below that is the best fit for your research. If you are not sure, read the appropriate sections before making your selection.

☒ Life sciences ☐ Behavioural & social sciences ☐ Ecological, evolutionary & environmental sciences

For a reference copy of the document with all sections, see nature.com/documents/nr-reporting-summary-flat.pdf

Life sciences study design

All studies must disclose on these points even when the disclosure is negative.

Sample size	Sample size are indicated in the figure legends. No statistical method was used to predetermine sample size. The sample size was based on previous studies in the field, e.g. Solis, et al. Cell. 2017;170(5):939-955.e24.
Data exclusions	No data were excluded from the analysis.
Replication	Quantification was carried out from a minimum number of 2-3 independent experiments. All replicates successfully reproduced the findings described in the manuscript.
Randomization	Randomization was not relevant to this study. Cells used throughout the study had to be differently treated and analyzed in parallel to minimize experimental variation. No human or animal subjects were used in this study. Randomization is not generally used in this field.
Blinding	The investigators were not blinded to allocation during experiments and outcome assessment. Blinding was not relevant to this study because assessment conditions were well controlled, results are quantitative in nature and did not require subjective judgment or interpretation. Blinding is not typically used in this field.

Reporting for specific materials, systems and methods

We require information from authors about some types of materials, experimental systems and methods used in many studies. Here, indicate whether each material, system or method listed is relevant to your study. If you are not sure if a list item applies to your research, read the appropriate section before selecting a response.

Materials & experimental systems

n/a	Involved in the study	
☐	☒	Antibodies
☐	☒	Eukaryotic cell lines
☒	☐	Palaeontology and archaeology
☒	☐	Animals and other organisms
☒	☐	Human research participants
☒	☐	Clinical data
☐	☐	Dual use research of concern

Methods

n/a	Involved in the study	
☒	☐	ChIP-seq
☒	☐	Flow cytometry
☒	☐	MRI-based neuroimaging

Antibodies

Antibodies used

Primary antibodies:
Mouse monoclonal against flotillin-2, clone 29, BD Biosciences (Cat# 610383), WB: 1/1000
Mouse monoclonal against GM130, clone 35, BD Biosciences (Cat# 610823), IF: 1/500
Rat monoclonal against HA-tag, clone 3F10, Roche, (Cat# 11867423001), IF: 1/500, WB: 1/2000
Mouse monoclonal against mRFP/DsRed2, no clone ID, Santa Cruz Biotechnology (Cat# sc-101526), WB: 1/250
Mouse monoclonal against GAPDH, clone 6C5, GeneTex (Cat# GTX28245), WB: 1/2000

Rabbit polyclonal against GFP, GeneTex (Cat# GTX113617), WB: 1/2000
Rabbit polyclonal against GFP, Takara (Cat# 632592), WB: 1/1000
Rabbit polyclonal against Flag-tag, Sigma-Aldrich (Cat# F7425), IF: 1/500; WB: 1/1000
Mouse monoclonal against α-tubulin, clone DM1A, Sigma-Aldrich (Cat# T6199), WB: 1/4000
Mouse monoclonal against His6-tag, no clone ID, Qiagen (Cat# 34650), IF: 1/500

Secondary antibodies:
Goat Anti-Mouse IgG (H+L) HRP-conjugated, Jackson ImmunoResearch (Cat# 115-035-062), WB: 1/5000
Goat Anti-Rabbit IgG (H+L) HRP-conjugated, Jackson ImmunoResearch (Cat# 111-035-144), WB: 1/5000
Goat Anti-Mouse IgG (H+L) Alexa Fluor 488 conjugated, Jackson ImmunoResearch (Cat# 111-545-144), IF: 1/500
Goat Anti-Mouse IgG (H+L) Cy3 conjugated, Jackson ImmunoResearch (Cat# 115-165-146), IF: 1/500
Goat Anti-Rat IgG (H+L) Cy3 conjugated, Jackson ImmunoResearch (Cat# 112-165-143), IF: 1/500
Goat Anti-Rabbit IgG (H+L) Cy5 conjugated, Jackson ImmunoResearch (Cat# 111-175-144), IF: 1/500

Validation

Flotillin-2, WB (mouse), AB_397766 (antibodyregistry.org), validated by manufacturer (https://www.bdbiosciences.com/us/reagents/research/antibodies-buffers/cell-biology-reagents/cell-biology-antibodies/purified-mouse-anti-flotillin-2-29flotillin-2/p/610383) and by us in previous siRNA- and shRNA-based knockdown studies (PMID: 17206938 and PMID: 23825023)
GM130, IF (mouse and human), AB_398142 (antibodyregistry.org), validated by manufacturer (https://www.bdbiosciences.com/us/reagents/research/antibodies-buffers/cell-biology-reagents/cell-biology-antibodies/purified-mouse-anti-gm130-35gm130/p/610823)
HA-tag, IF (peptide tag), AB_390918 (antibodyregistry.org), validated by manufacturer (https://www.sigmaaldrich.com/catalog/product/roche/roahaha) and by us in negative IF signals of non-transfected cells (PMID: 23825023)
mRFP/DsRed, WB (fluorescent protein), AB_1562589 (antibodyregistry.org), validated by manufacturer (https://www.scbt.com/de/p/dsred2-antibody-25) and by us in negative WB signals of non-transfected cells (this study)
GAPDH, WB (mouse), AB_370675 (antibodyregistry.org), validated by manufacturer (https://www.genetex.com/Product/Detail/GAPDH-antibody-6C5/GTX28245)
GFP, WB (fluorescent protein), AB_1950371 (antibodyregistry.org), validated by manufacturer (https://www.genetex.com/Product/Detail/GFP-antibody/GTX113617) and by us in negative WB signals of non-transfected cells
GFP, WB (fluorescent protein), AB_2336883 (antibodyregistry.org), validated by manufacturer (https://www.takarabio.com/products/antibodies-and-elisa/fluorescent-protein-antibodies/green-fluorescent-protein-antibodies?catalog=632592)
Flag-tag, IF and WB (peptide tag), AB_439687 (antibodyregistry.org), validated by manufacturer (https://www.sigmaaldrich.com/CH/de/product/sigma/f7425) and by us in negative WB and IF signals of non-transfected cells (this study)
α-tubulin, WB (mouse), AB_477583 (antibodyregistry.org), validated by manufacturer (https://www.sigmaaldrich.com/CH/de/product/sigma/t6199)
His6-tag, IF (peptide tag), AB_2687898 (antibodyregistry.org), validated by manufacturer (https://www.qiagen.com/us/products/discovery-and-translational-research/protein-purification/tagged-protein-expression-purification-detection/anti-his-antibodies-bsa-free) and by us in negative IF signals of non-transfected cells (PMID: 24560274)

Eukaryotic cell lines

Policy information about cell lines

Cell line source(s)

Mouse neuroblastoma Neuro-2a were obtained from ATCC (CCL-131)
Human epithelial HeLa were obtained from ATCC (CCL-2)
Drosophila Schneider-2 (S2) cells were from Invitrogen (R690-07)

Authentication

None of the cell lines used were authenticated

Mycoplasma contamination

All lines were routinely checked and tested negative for mycoplasma contamination

Commonly misidentified lines
(See ICLAC register)

No commonly misidentified cell lines were used

Dual use research of concern

Policy information about dual use research of concern

Hazards

Could the accidental, deliberate or reckless misuse of agents or technologies generated in the work, or the application of information presented in the manuscript, pose a threat to:

No	Yes	
☒	☐	Public health
☒	☐	National security
☒	☐	Crops and/or livestock
☒	☐	Ecosystems
☒	☐	Any other significant area

Experiments of concern

Does the work involve any of these experiments of concern:

No	Yes	
☒	☐	Demonstrate how to render a vaccine ineffective
☒	☐	Confer resistance to therapeutically useful antibiotics or antiviral agents
☒	☐	Enhance the virulence of a pathogen or render a nonpathogen virulent
☒	☐	Increase transmissibility of a pathogen
☒	☐	Alter the host range of a pathogen
☒	☐	Enable evasion of diagnostic/detection modalities
☒	☐	Enable the weaponization of a biological agent or toxin
☒	☐	Any other potentially harmful combination of experiments and agents
