# Supplementary material for: Local and substrate-specific S-palmitoylation determines subcellular localization of Gαo
Source: Nat Commun. 2022 Apr 19;13:2072. doi: 10.1038/s41467-022-29685-8 (PMC9018777; doi:10.1038/s41467-022-29685-8)
Supplement: Supplementary file 12 — Source Data [file 41467_2022_29685_MOESM12_ESM.zip › Uncropped WBs.pdf]

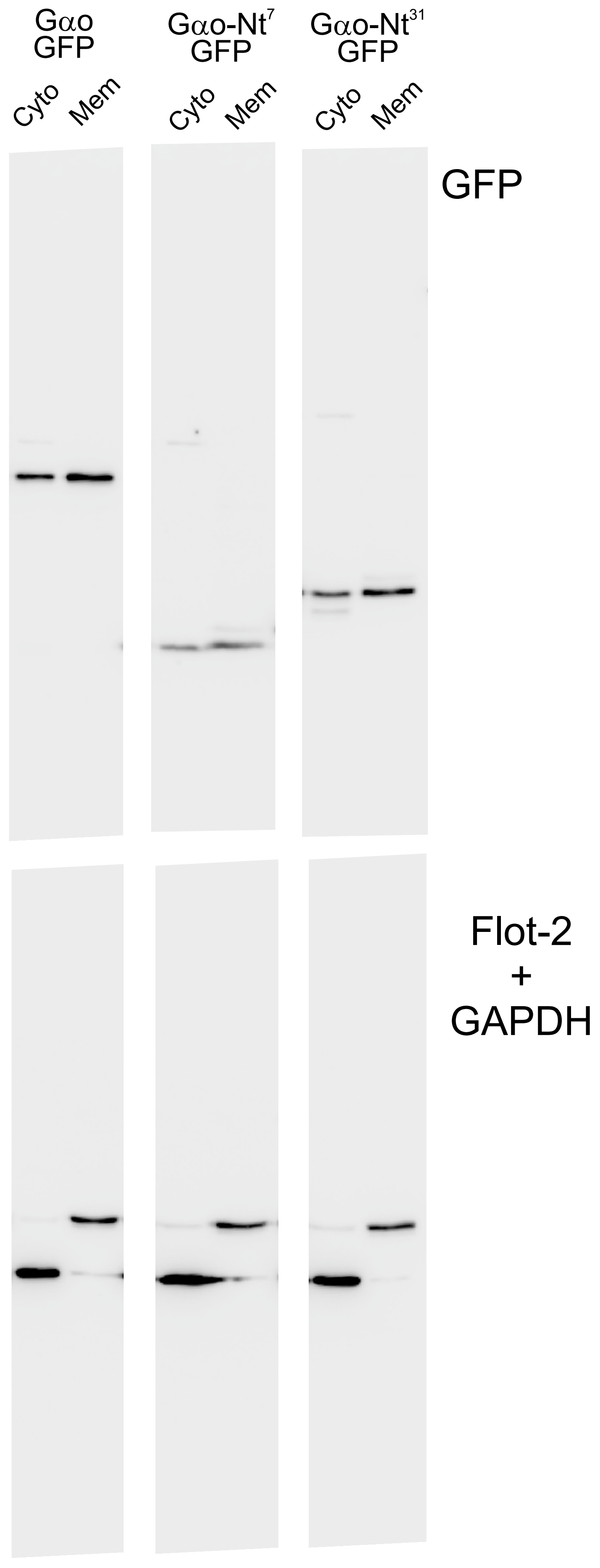

Figure 1g_Solis et al.

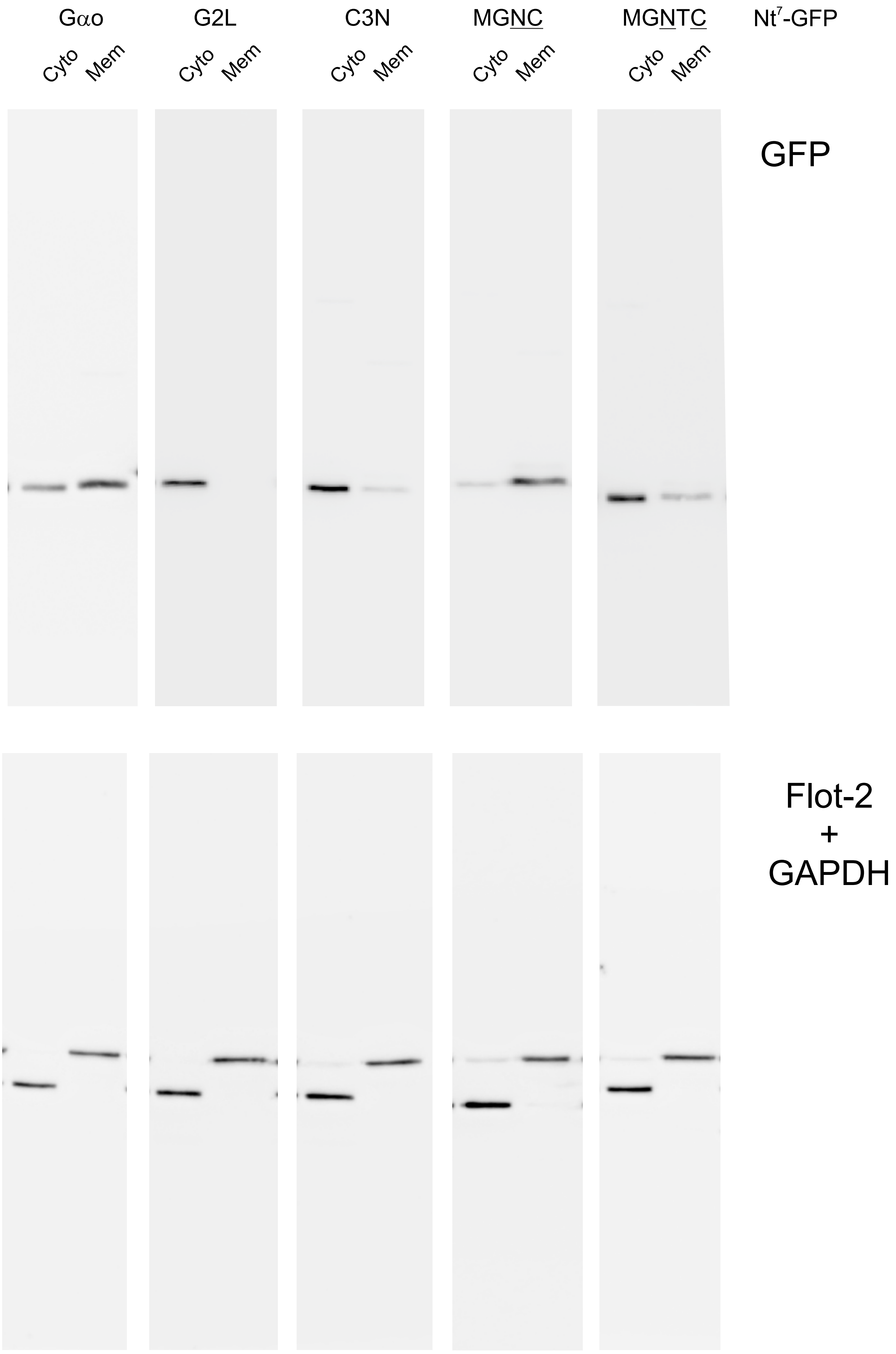

Figure 1k_Solis et al.

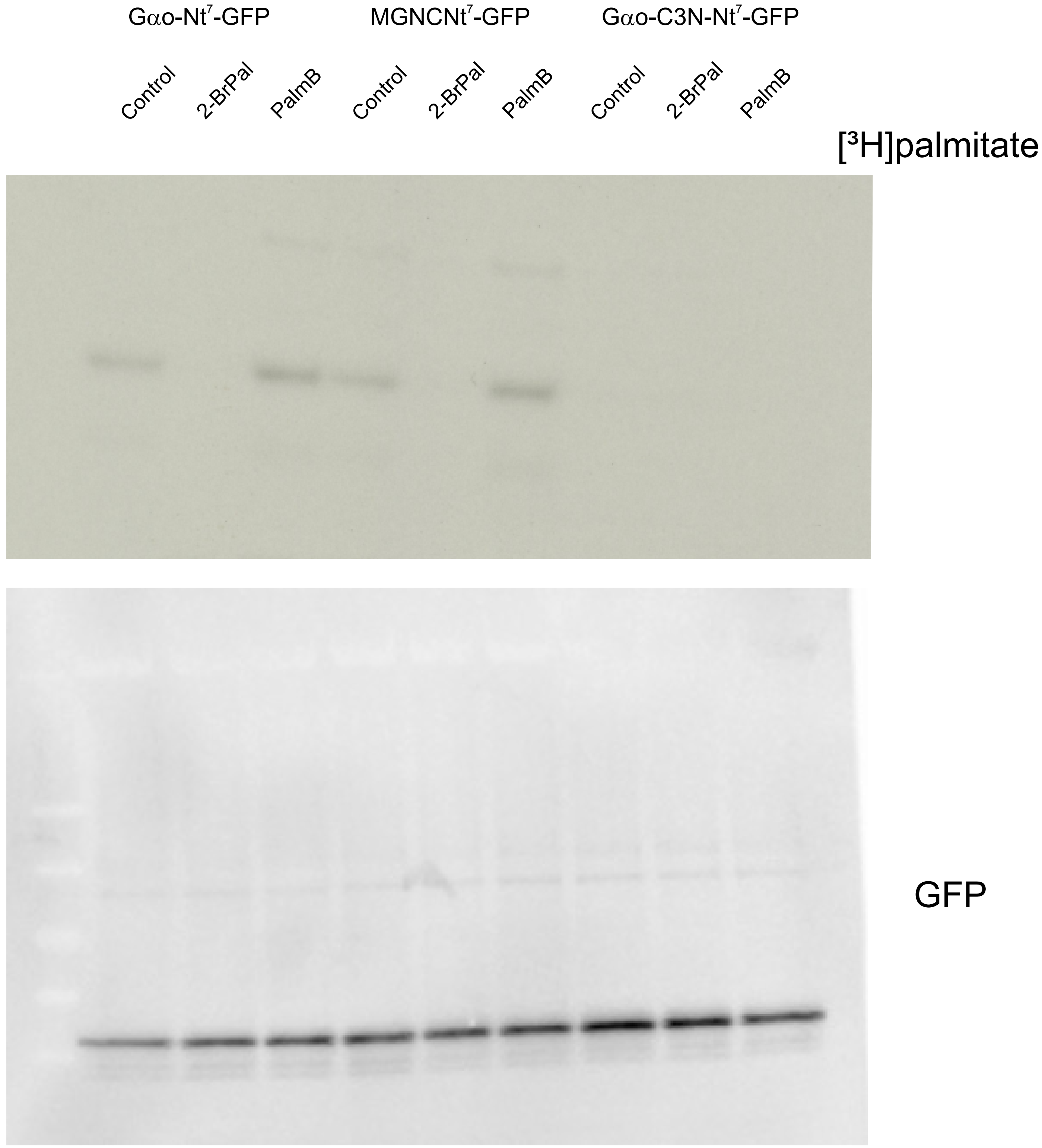

Figure 1o_Solis et al.

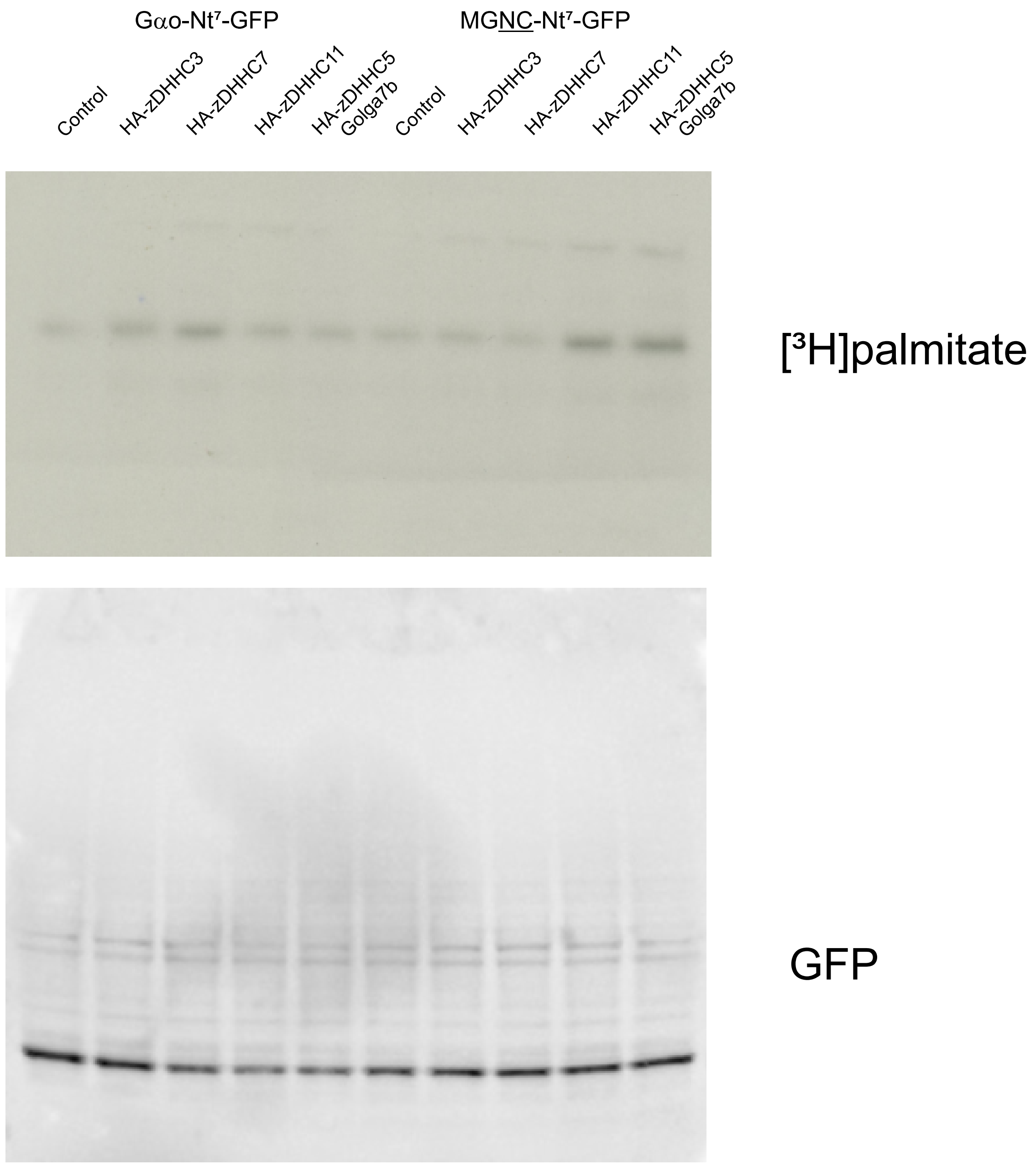

Figure 7m_Solis et al.

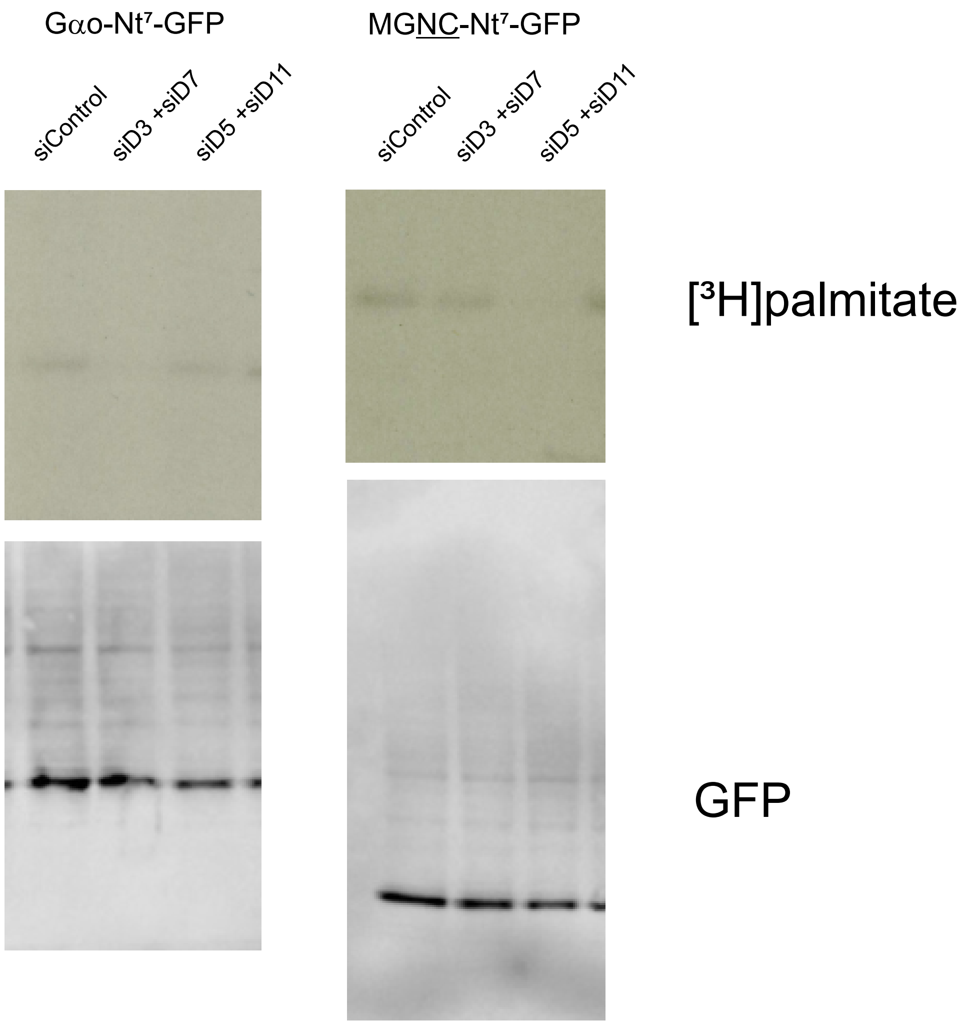

Figure 8h_Solis et al.

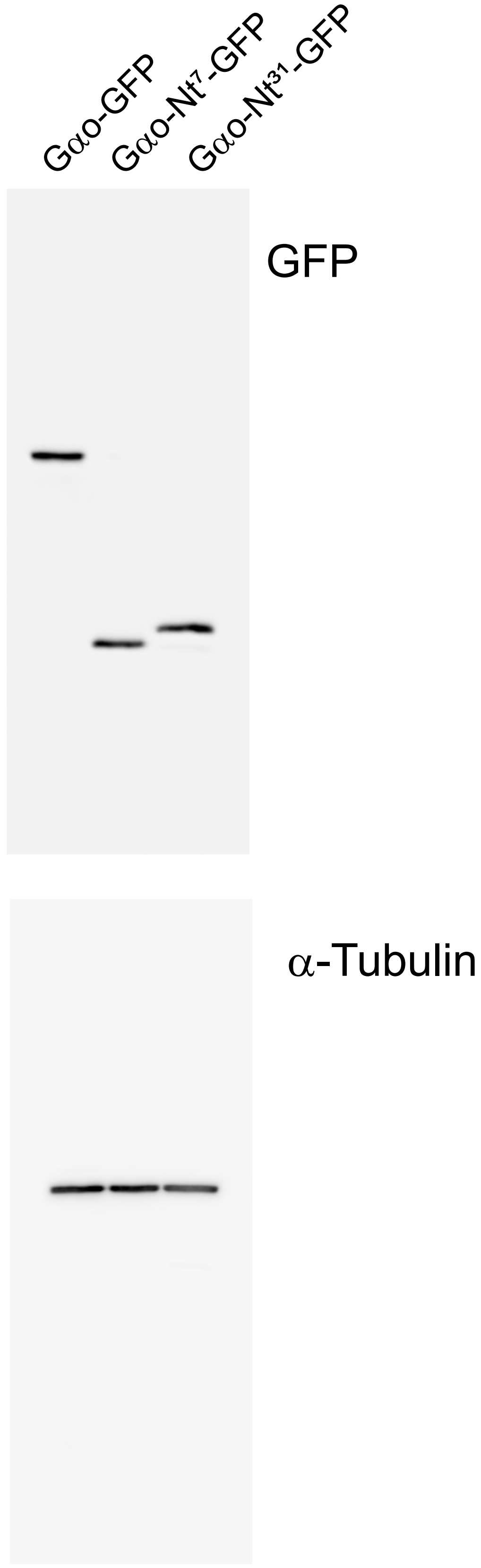

Supp. Figure 1g_Solis et al.

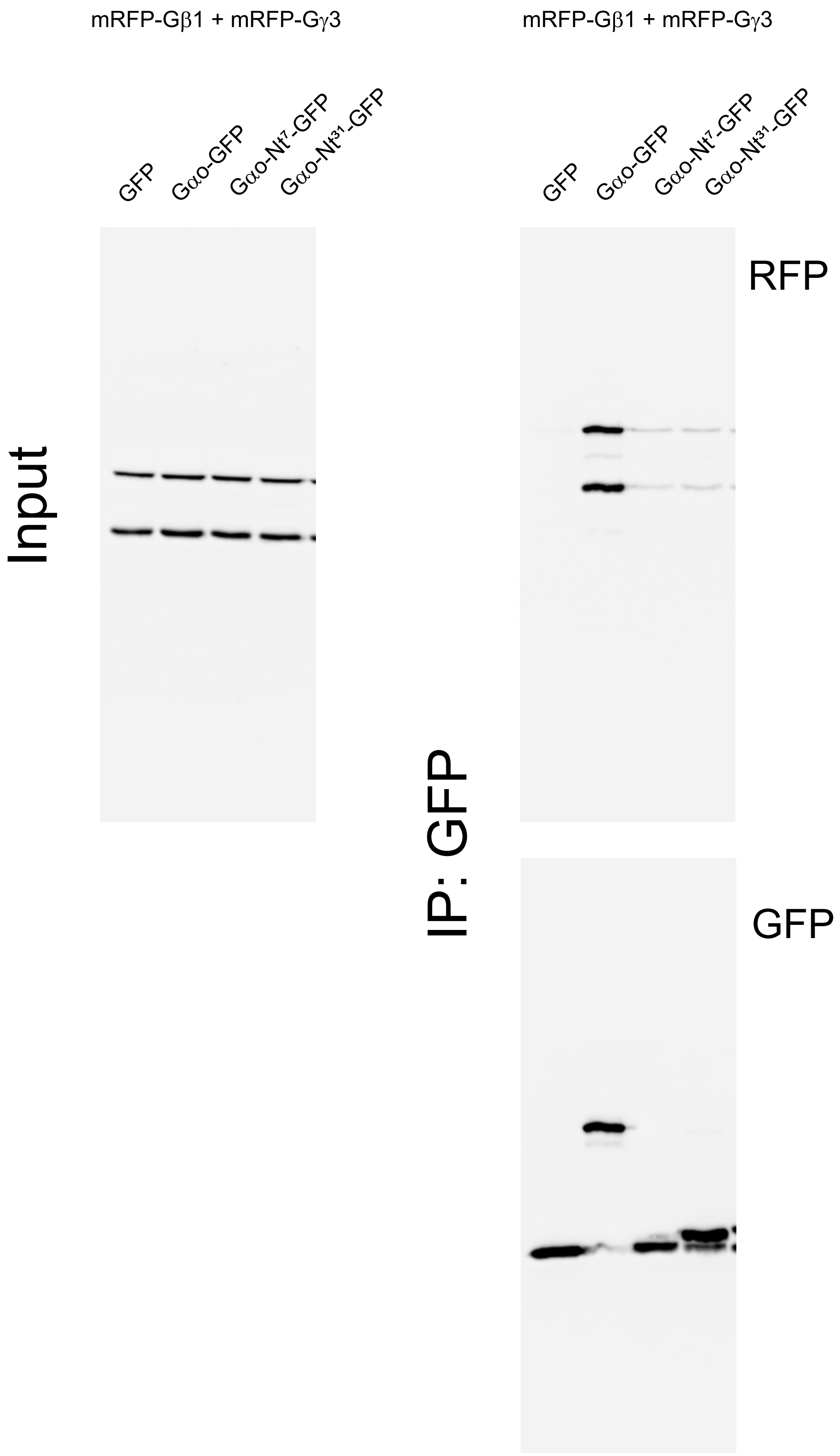

Supp. Figure 1j_Solis et al.

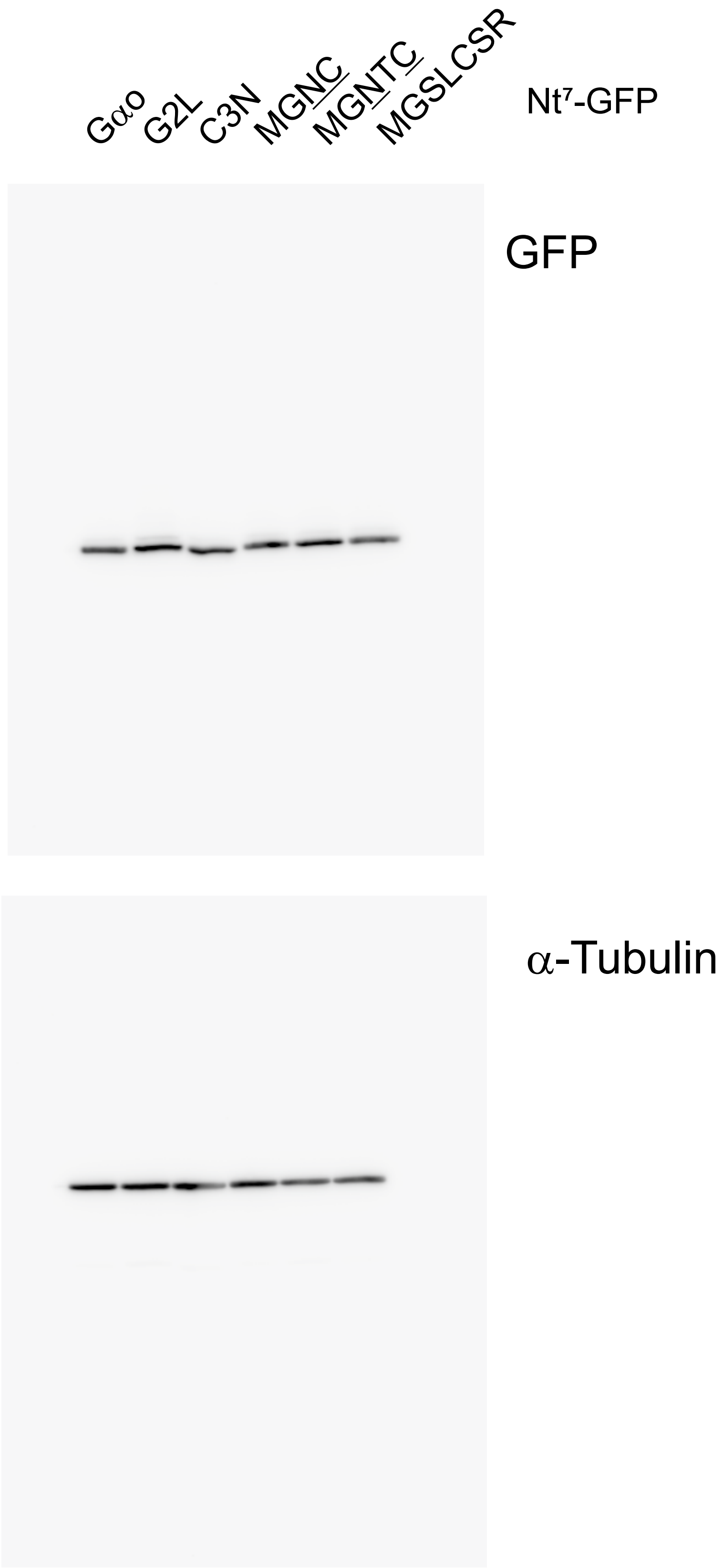

Supp. Figure 1k_Solis et al.

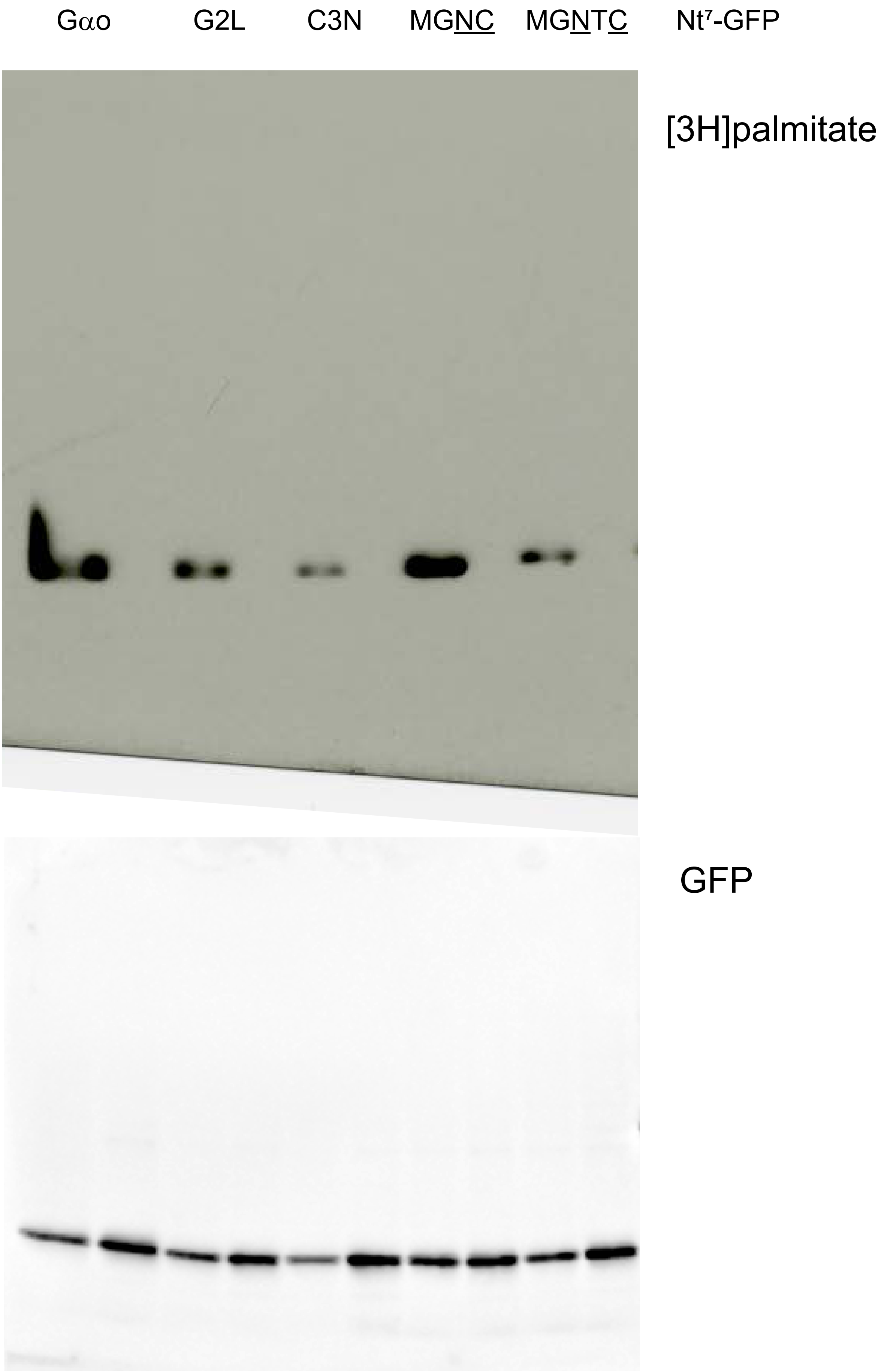

Supp. Figure 1m_Solis et al.

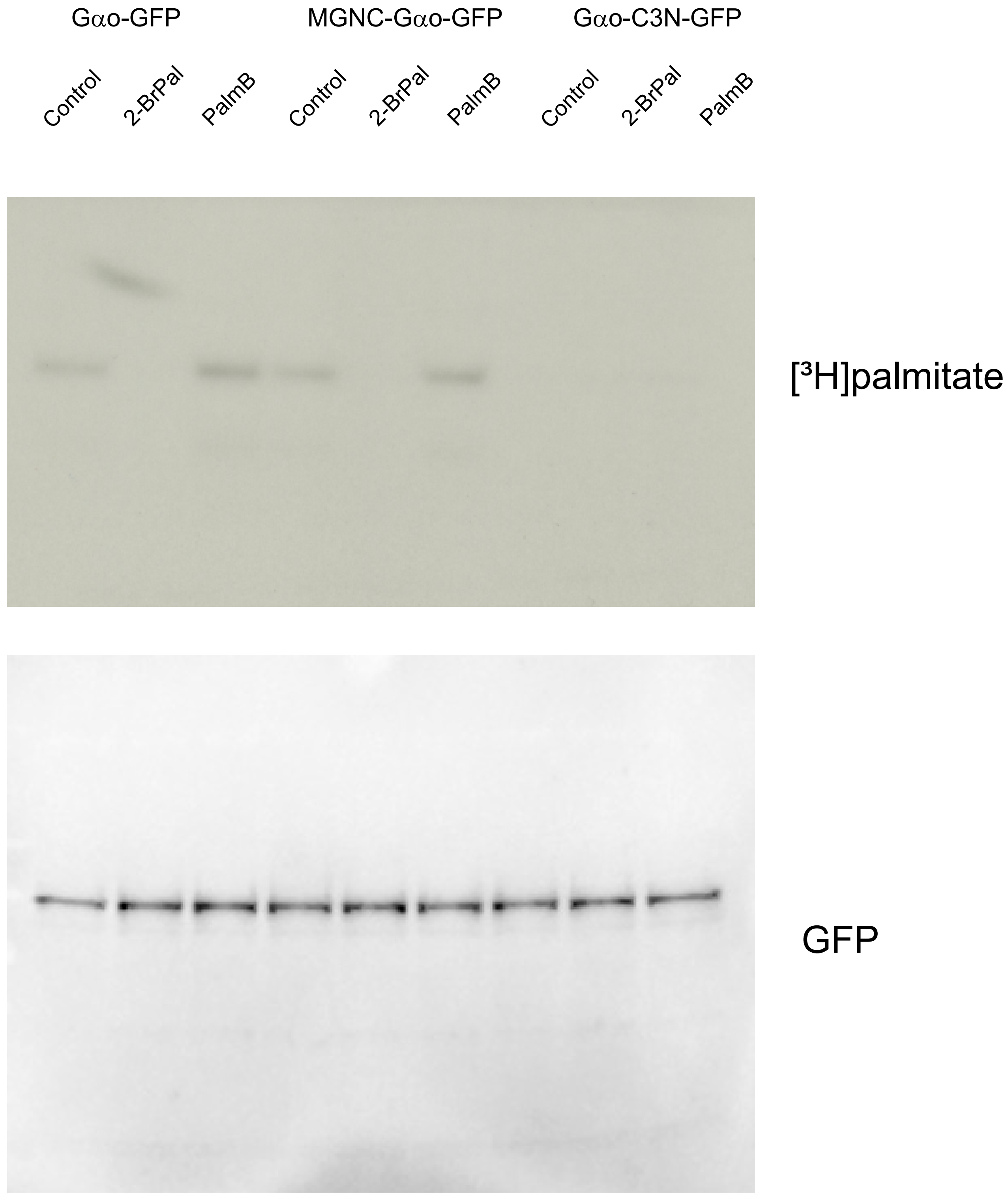

Supp. Figure 1o_Solis et al.

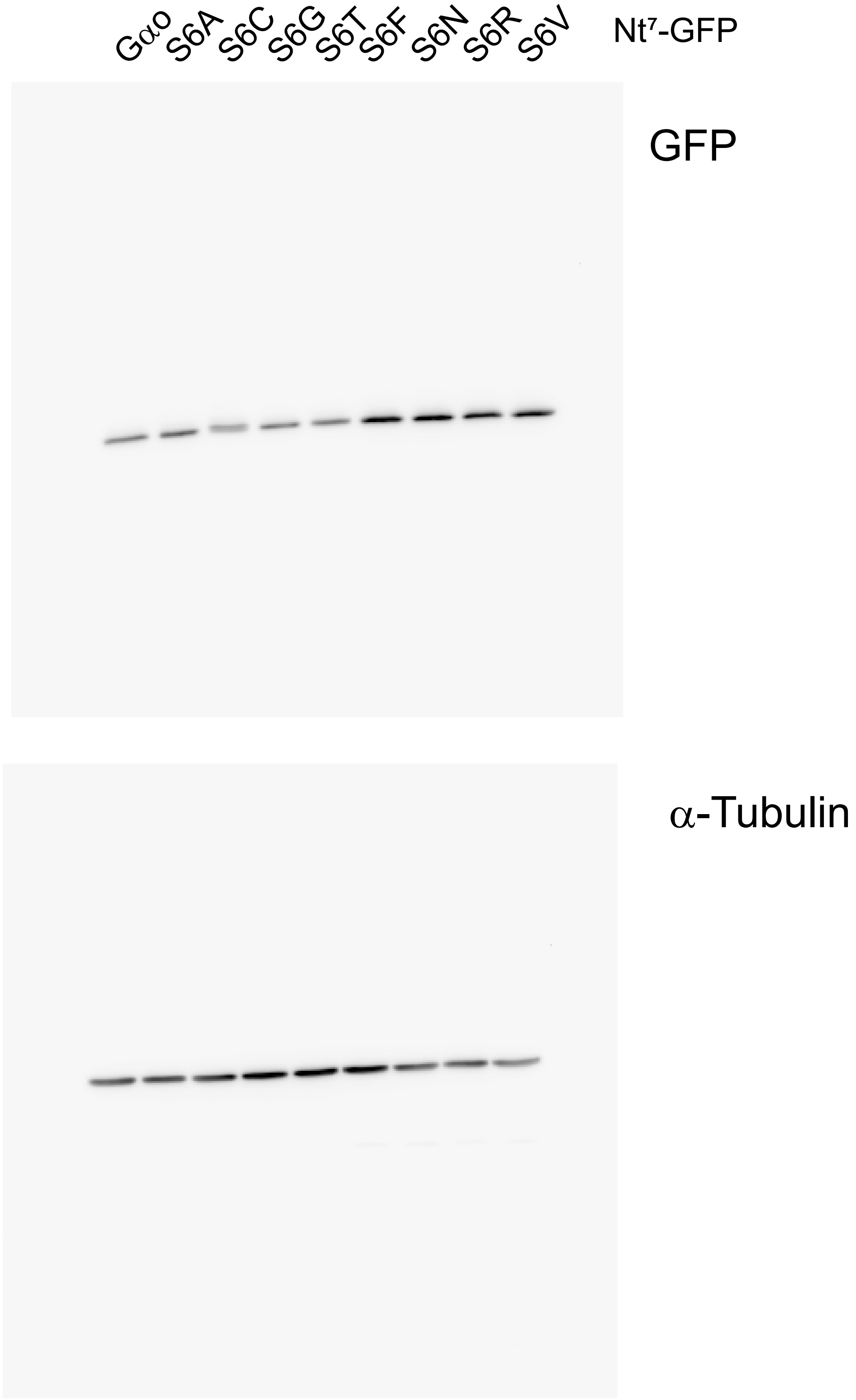

Supp. Figure 2a_Solis et al.

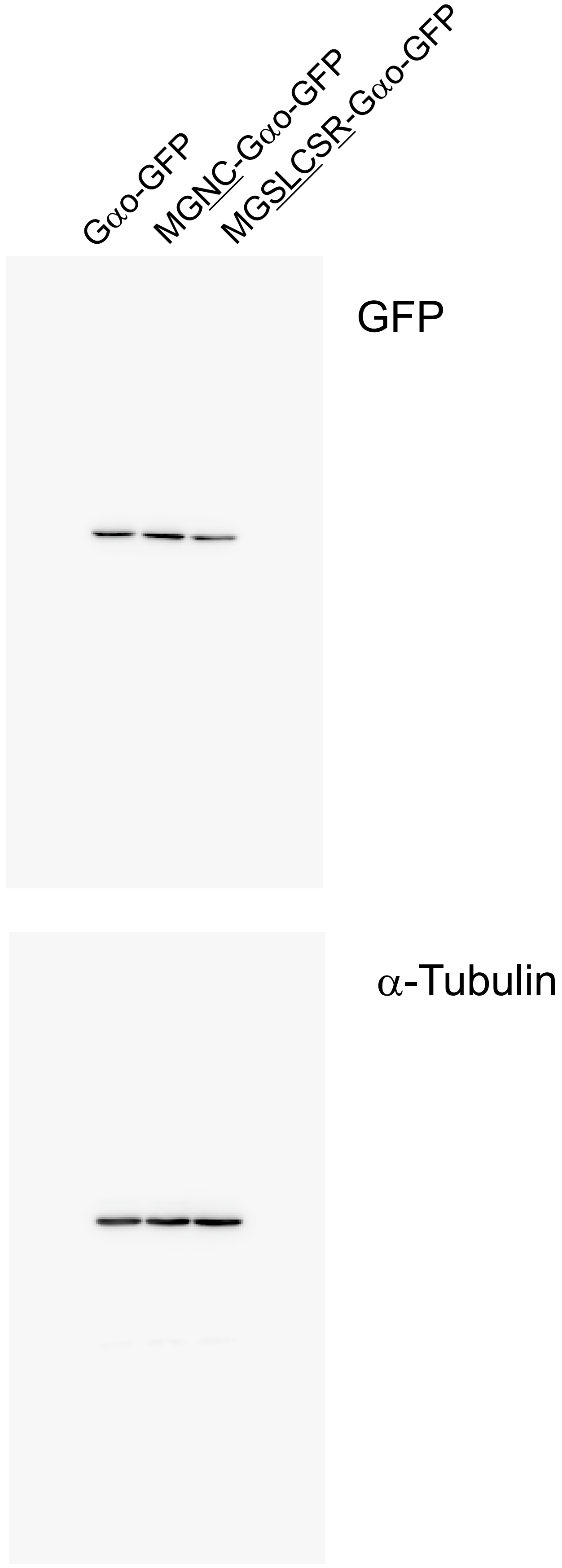

Supp. Figure 2g_Solis et al.

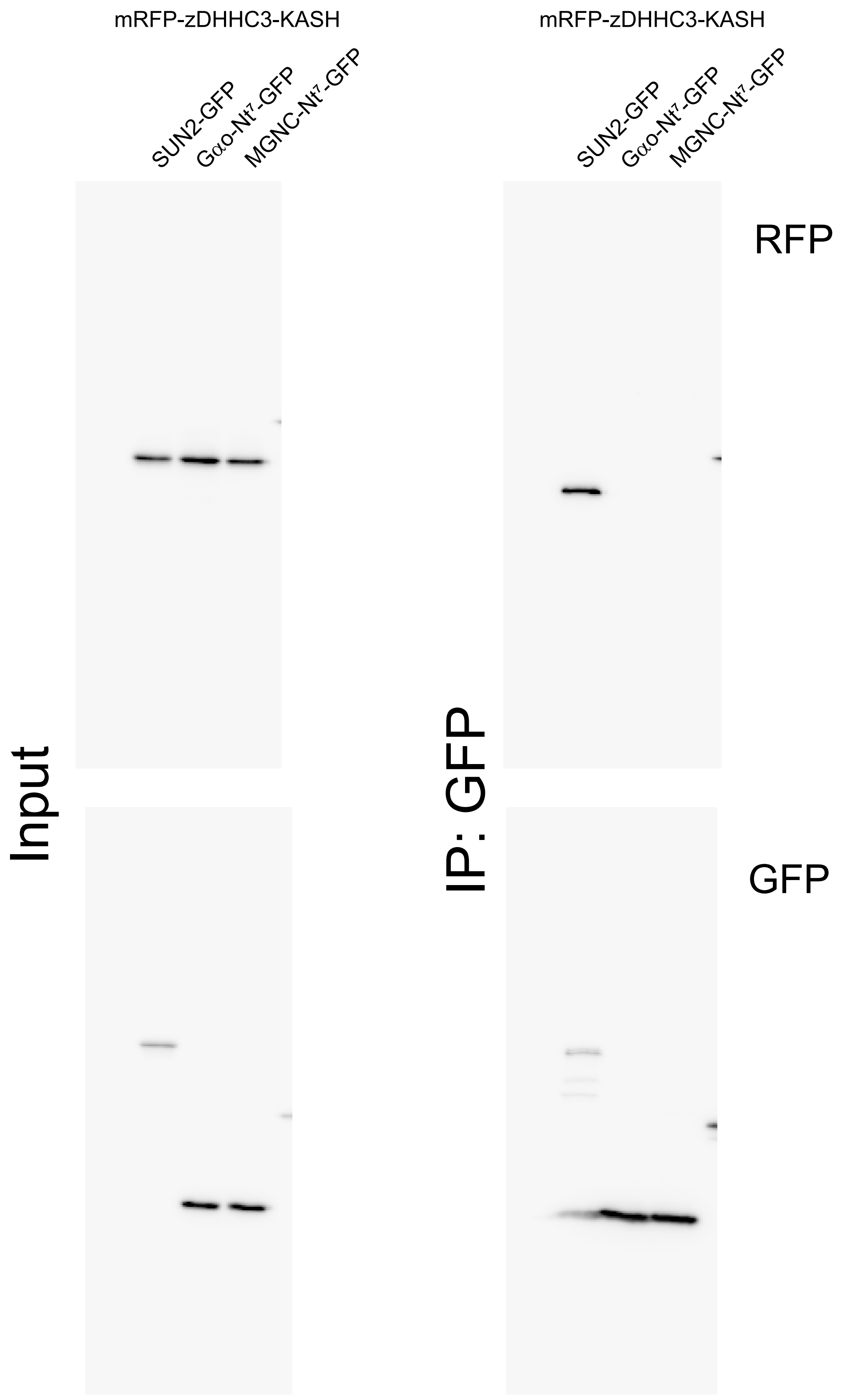

Supp. Figure 6d_Solis et al.

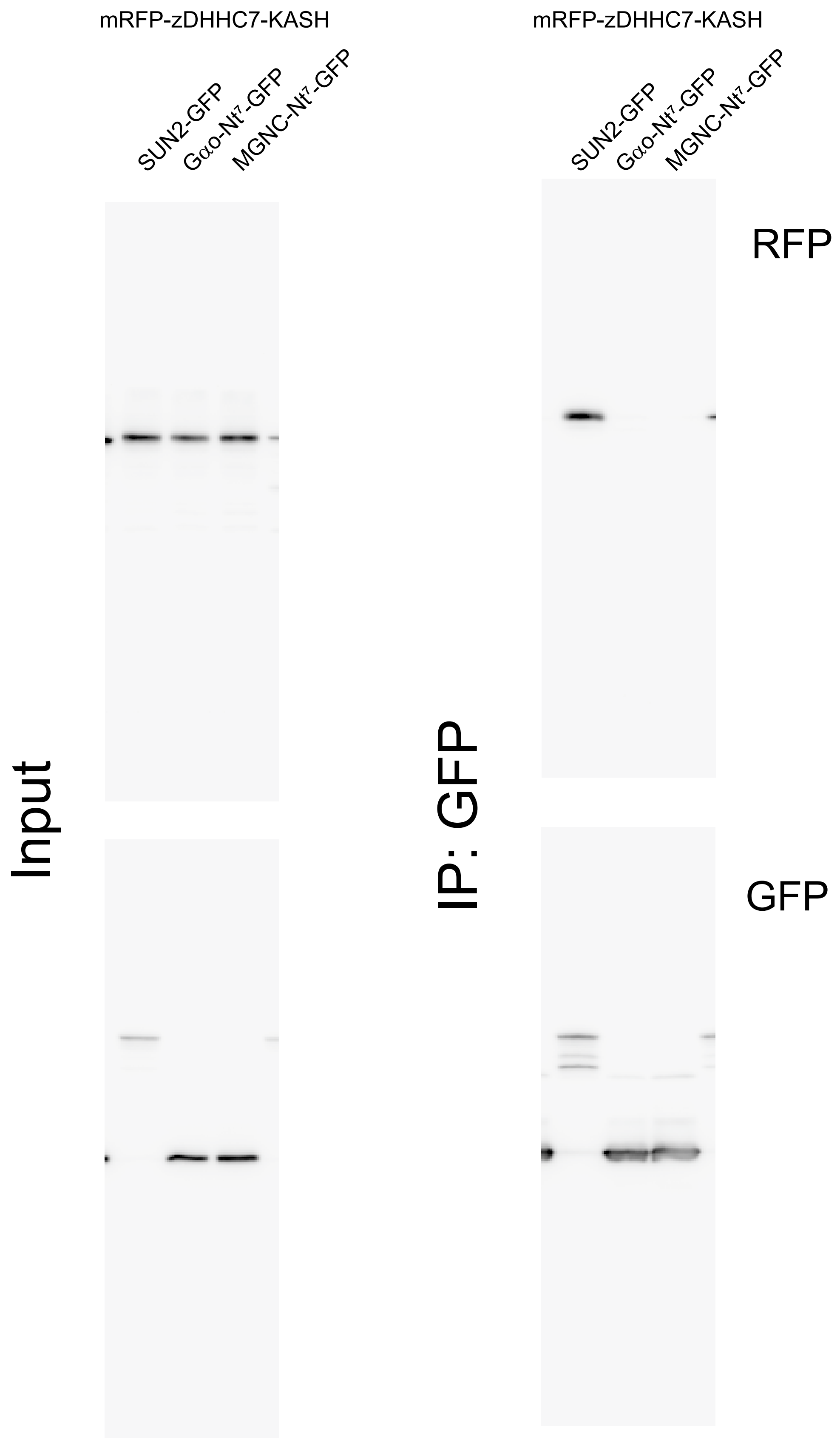

Supp. Figure 6e_Solis et al.

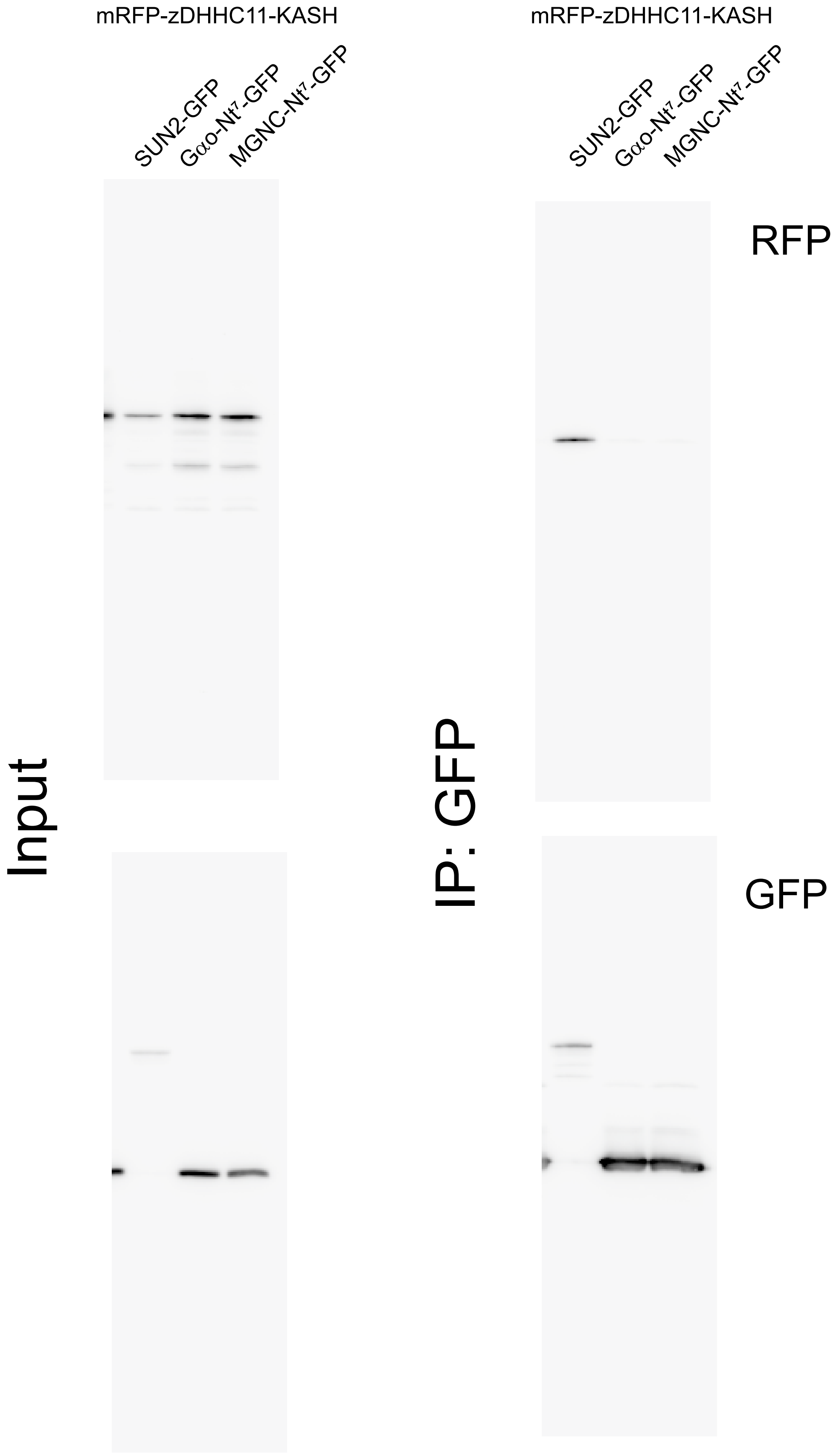

Supp. Figure 6f_Solis et al.

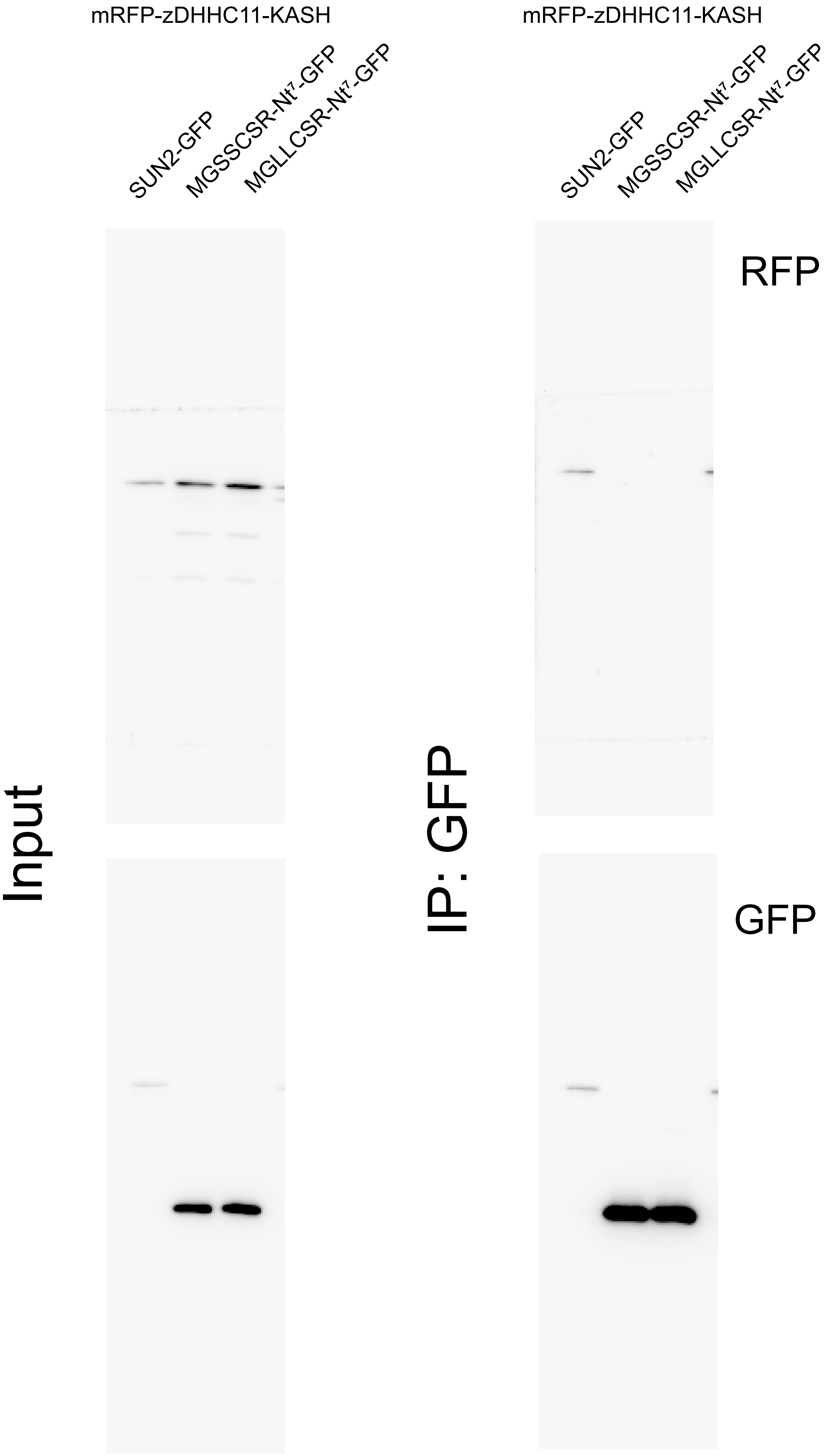

Supp. Figure 6g_Solis et al.

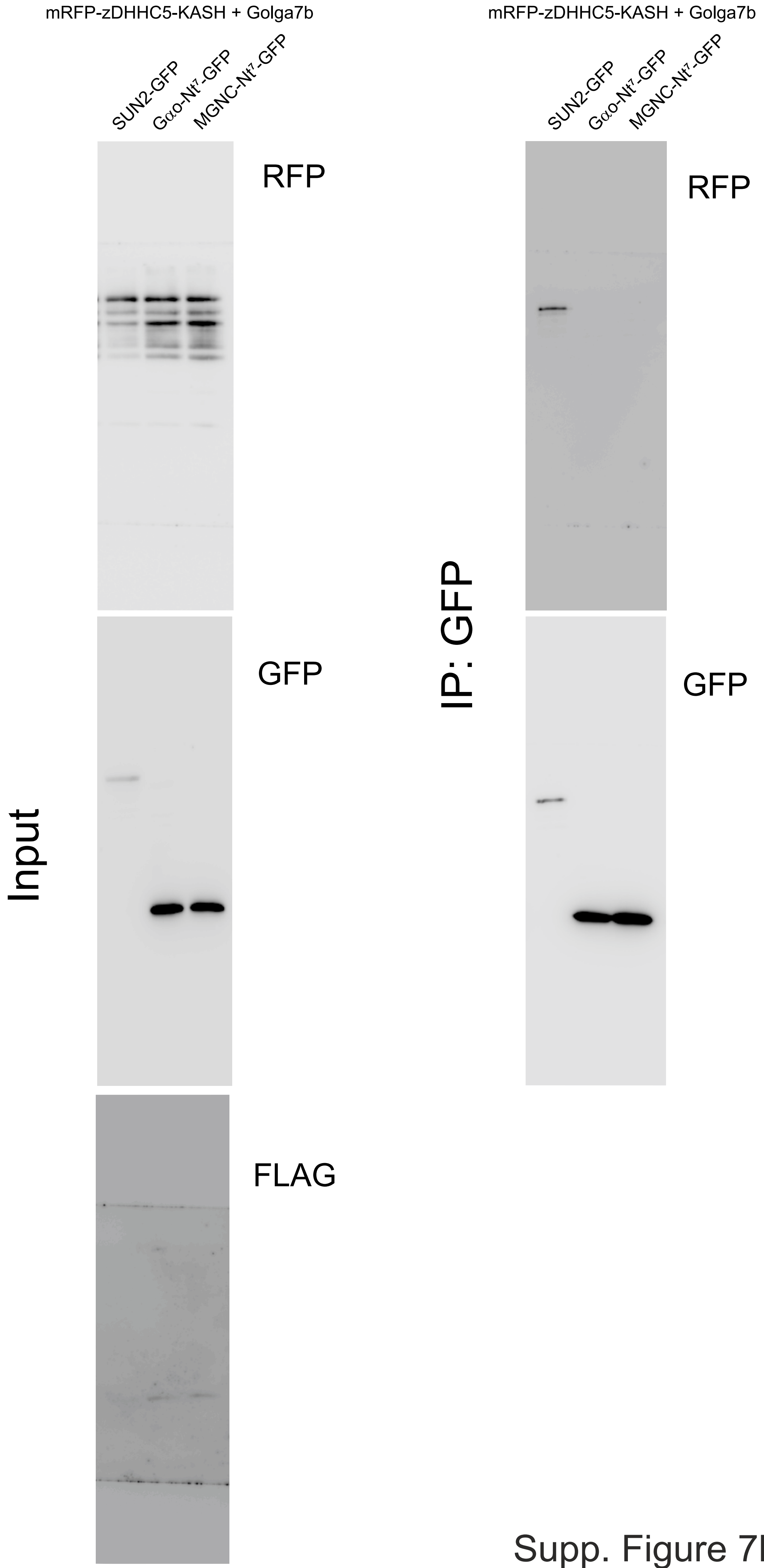

Supp. Figure 7h_Solis et al.

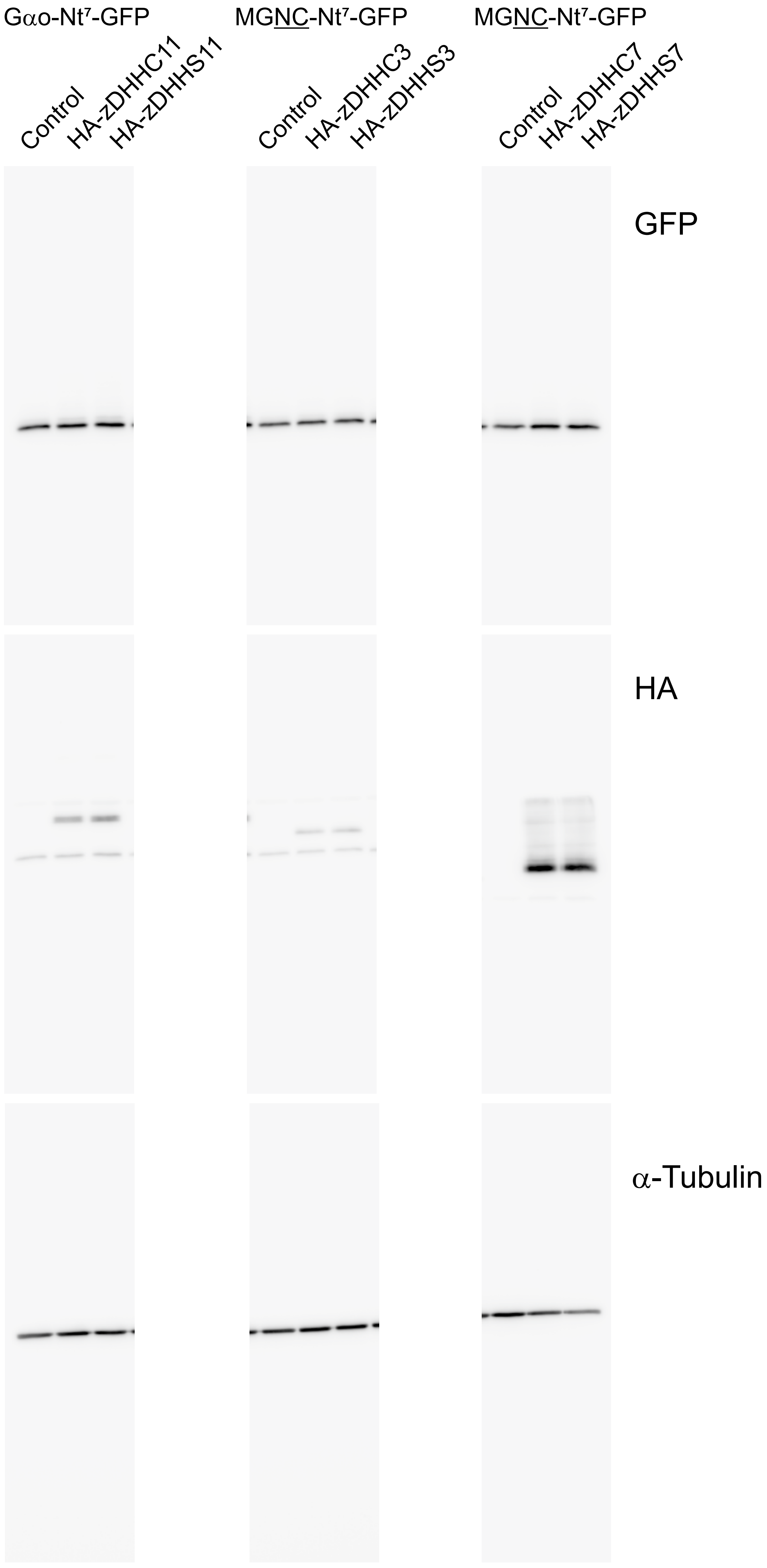

Supp. Figure 8c-e_Solis et al.

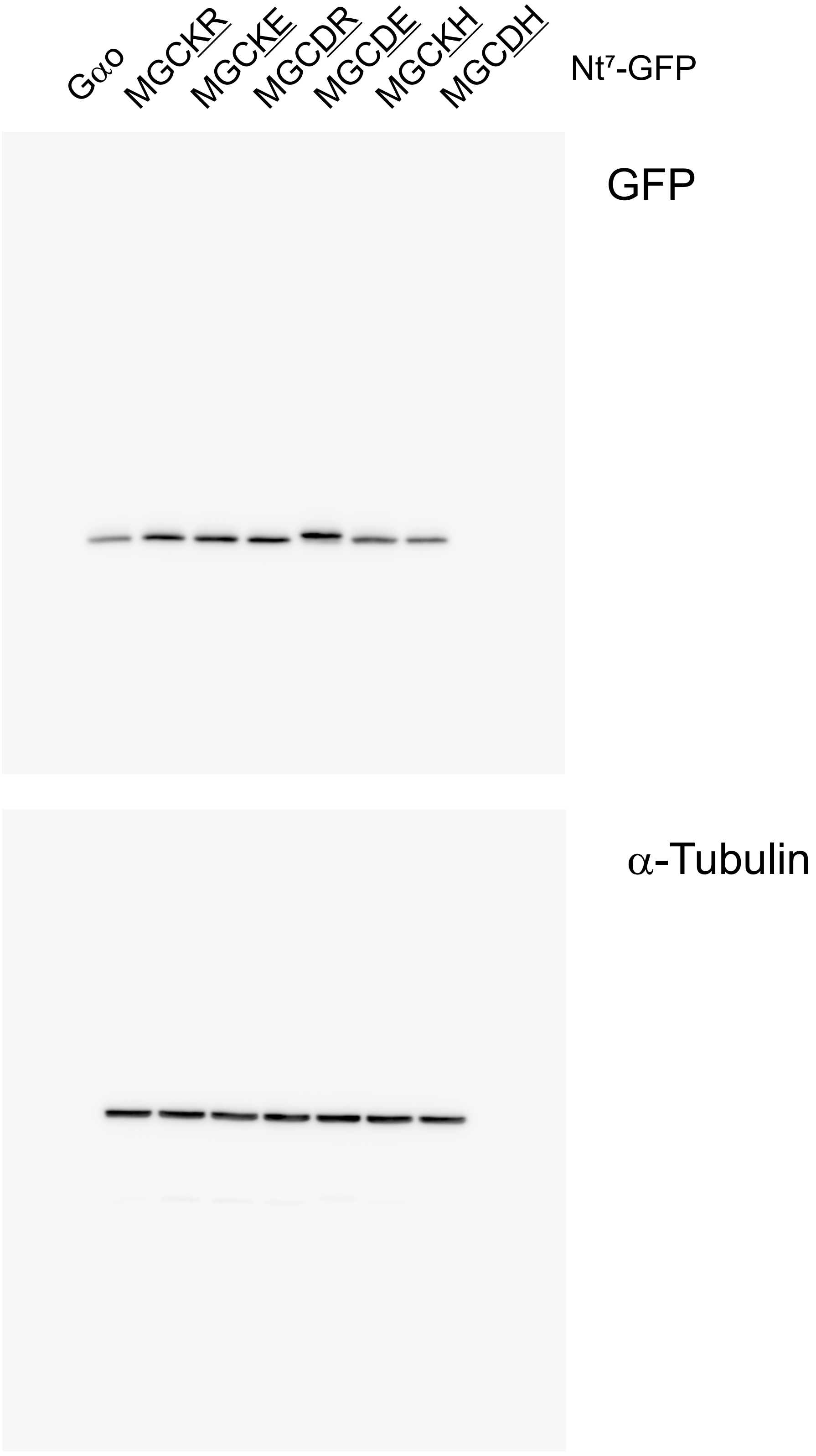

Supp. Notes Figure 1g_Solis et al.
